# Ischemia-Selective Cardioprotection by Malonate for Ischemia/Reperfusion Injury

**DOI:** 10.1161/CIRCRESAHA.121.320717

**Published:** 2022-08-12

**Authors:** Hiran A. Prag, Dunja Aksentijevic, Andreas Dannhorn, Abigail V. Giles, John F. Mulvey, Olga Sauchanka, Luping Du, Georgina Bates, Johannes Reinhold, Duvaraka Kula-Alwar, Zhelong Xu, Luc Pellerin, Richard J. A. Goodwin, Michael P. Murphy, Thomas Krieg

**Affiliations:** 1 Department of Medicine, University of Cambridge, Cambridge, CB2 0QQ, UK; 2 MRC Mitochondrial Biology Unit, University of Cambridge, Cambridge, CB2 0XY, UK; 3 Centre for Biochemical Pharmacology, William Harvey Research Institute, Barts and The London School of Medicine and Dentistry, Queen Mary University of London, London, UK; 4 Imaging and Data Analytics, Clinical Pharmacology and Safety Sciences, R&D, AstraZeneca, Cambridge, UK; 5 Laboratory of Cardiac Energetics, National Heart, Lung and Blood Institute, Bethesda, MD, USA; 6 Department of Physiology and Pathophysiology, Tianjin Medical University, Tianjin 300070, China; 7 Faculty of Medicine and Health Sciences, University of East Anglia, Norwich Research Park, Norwich NR4 7TJ; 8 Département de Physiologie, Université de Lausanne, 7 Rue du Bugnon, 1005 Lausanne, Switzerland; 9 Centre de Résonance Magnétique des Systèmes Biologiques, UMR5536 CNRS, LabEx TRAIL-IBIO, Université de Bordeaux, Bordeaux Cedex, 33760, France; 10 Inserm U1313, Université et CHU de Poitiers, Poitiers Cedex, 86021, France; 11 Institute of Infection, Immunity and Inflammation, College of Medical, Veterinary and Life Sciences, University of Glasgow, Glasgow G12 8QQ, UK

**Keywords:** ischemia/reperfusion injury, myocardial infarction, mitochondria, malonate, drug delivery, reactive oxygen species, succinate

## Abstract

**Background:**

Inhibiting succinate dehydrogenase (SDH), with the competitive inhibitor malonate, has shown promise in ameliorating ischemia/reperfusion injury. However, key for translation to the clinic is understanding the mechanism of malonate entry into cells to enable inhibition of SDH, its mitochondrial target, as malonate itself poorly permeates cellular membranes. The possibility of malonate selectively entering the at-risk heart tissue on reperfusion, however, remains unexplored.

**Methods:**

C57BL/6J mice, C2C12 and H9c2 myoblasts and HeLa cells were used to elucidate the mechanism of selective malonate uptake into the ischemic heart upon reperfusion. Cells were treated with malonate while varying pH or together with transport inhibitors. Mouse hearts were either perfused *ex vivo* (Langendorff) or subjected to *in vivo* left anterior descending coronary artery ligation as models of ischemia/reperfusion injury. Succinate and malonate levels were assessed by LC-MS/MS, *in vivo* by mass spectrometry imaging and infarct size by TTC staining.

**Results:**

Malonate was robustly protective against cardiac IR injury, but only if administered at reperfusion and not when infused prior to ischemia. The extent of malonate uptake into the heart was proportional to the duration of ischemia. Malonate entry into cardiomyocytes *in vivo* and *in vitro* was dramatically increased at the low pH (~6.5) associated with ischemia. This increased uptake of malonate was blocked by selective inhibition of monocarboxylate transporter 1 (MCT1). Reperfusion of the ischemic heart region with malonate led to selective SDH inhibition in the at-risk region. Acid-formulation greatly enhances the cardioprotective potency of malonate.

**Conclusions:**

Cardioprotection by malonate is dependent on its entry into cardiomyocytes. This is facilitated by the local decrease in pH that occurs during ischemia, leading to its selective uptake upon reperfusion into the at-risk tissue, via MCT1. Thus, malonate’s preferential uptake in reperfused tissue means it is an at-risk tissue-selective drug that protects against cardiac ischemia/reperfusion injury.

## Graphical abstract

**Graphical abstract:**
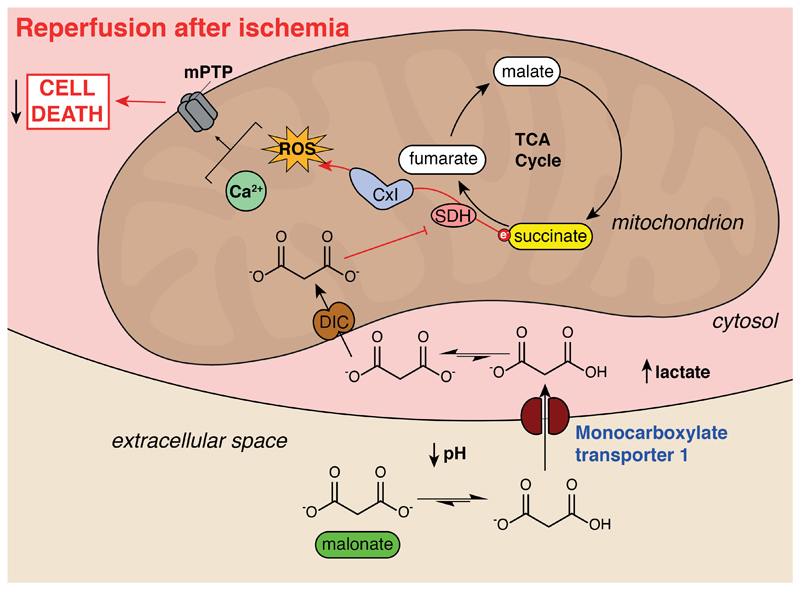

## Introduction

Myocardial infarction (MI) and the consequential heart failure (HF) is a leading cause of mortality.^1,2^ Early reperfusion of the ischemic myocardium by primary percutaneous coronary intervention (PPCI) is essential to minimize cardiomyocyte death.^3,4^ However, paradoxically, the reintroduction of oxygen into the cardiac tissue accelerates cardiomyocyte death by ischemia/reperfusion (IR) injury thereby increasing tissue injury.^5–9^ While patients now often survive their initial infarct, IR injury is a major driver of post-MI heart failure.^1,5,10,11^ Pharmacological interventions to reduce IR injury have remained elusive, likely as a result of a poor understanding of the pathology.^12–16^ Recent research on mitochondrial metabolic changes during IR injury has opened up new therapeutic opportunities.^1,5,10,11,13,15,17^

During ischemia, the mitochondrial metabolite succinate accumulates dramatically in ischemic tissue, reaching millimolar concentrations.^18–20^ The accumulation of succinate and degradation of purine nucleotides are now seen as hallmarks of ischemia conserved in many species, including humans.^18,19,21^ During reperfusion, the accumulated succinate is rapidly oxidized by the mitochondrial respiratory chain enzyme succinate dehydrogenase (SDH), driving reactive oxygen species (ROS) production by reverse electron transport (RET) at mitochondrial complex I.^13,18,22,23^ The production of ROS by RET upon reperfusion induces the mitochondrial permeability transition pore (mPTP) opening, initiating the significant cell death associated with IR injury. ^13,18,22^

**Figure 1:**
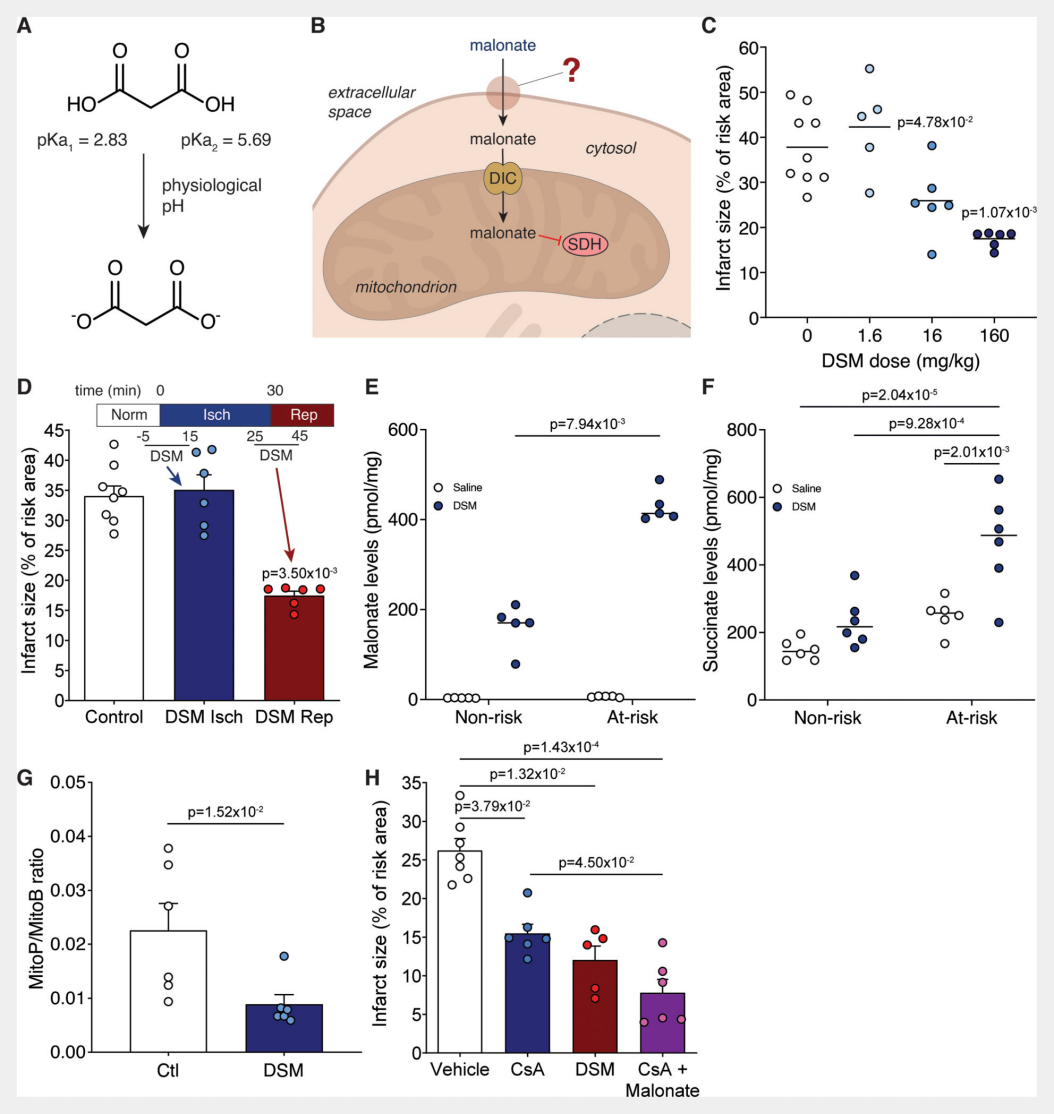
Malonate is cardioprotective only when given at reperfusion. **A**, Malonate predominantly carries two negatively charged dicarboxylates at physiological pH. **B**, Schematic of the barriers to effective malonate delivery. **C**, Infarct size in murine LAD model with infusion of varying doses of DSM (0, 1.6, 16 or 160 mg/kg; n=9, 5, 6, 6 respectively) infused at reperfusion after 30 min ischemia, quantified by TTC staining. **D**, Infarct size in murine LAD model with infusion of DSM (160 mg/kg) at ischemia or at reperfusion (mean ± S.E.M., n=8 (Control), 6 (DSM isch and DSM rep) biological replicates. **E** and **F**, Levels of malonate (**E**) and succinate (**F**) in non-risk and at-risk tissue after 1 min reperfusion with either saline or DSM (160 mg/kg) after 30 min ischemia (mean ± S.E.M., n=5 (**E**), 6 (**F**) biological replicates). Statistics: Kruskal-Wallis with Dunn’s post hoc test (**C** to **E**), two-way ANOVA with Tukey’s post hoc test (**F**). **G**, MitoP/B ratio in at-risk heart tissue from LAD model after 30 min ischemia and 15 min reperfusion with either saline (Ctl) or DSM (160 mg/kg) infusion (mean ± S.E.M, n=6 biological replicates, statistics: unpaired, two-tailed Mann-Whitney U test) **H**, Infarct size in murine LAD model with infusion of vehicle (ethanol/Cremphor EL in saline) ± cyclosporin A (10 mg/kg) or DSM (160 mg/kg) at reperfusion (mean ± S.E.M, n=7 (vehicle), 6 (CsA, CsA + Malonate), 5 (DSM) biological replicates, statistics: Kruskal-Wallis with Dunn’s post hoc test).

This mechanism of RET-derived ROS driving IR injury was supported recently by the finding that a mouse model carrying a mutation (mtDNA *ND6* G14600A leading to ND6 P25L substitution) that inactivates RET, is protected from cardiac IR injury.^22^ Blocking succinate accumulation using dimethyl malonate ester (DMM), a prodrug of the SDH inhibitor malonate, prior to ischemia was also cardioprotective.^18,24,25^ However, DMM was not protective when infused at the clinically-relevant point of reperfusion, due to its slow release of malonate; this could be circumvented using more rapidly hydrolysed malonate ester prodrugs.^25^ The cell-permeable malonate esters were developed because malonate was assumed to be cell membrane impermeant. Therefore, it was surprising that malonate itself, administered as disodium malonate (DSM), was protective against cardiac IR injury in mouse *ex vivo* and pig *in vivo*.^26,27^ Malonate is a dicarboxylate carrying two negative charges at physiological pH (Figure 1A) and has poor membrane permeability. This is highlighted by the need for high concentrations and extended incubation times to see malonate-dependent effects *in vitro*.^28^ Therefore, the mechanism of DSM cardioprotection and thus its potential for translation to the clinic, is unclear (Figure 1B).

Here, we set out to understand the mechanism by which DSM is cardioprotective. We found that under physiological pH (~7.4), very high concentrations of malonate were required for SDH inhibition in cells. However, at lower pH malonate uptake into cells and the heart was considerably increased. Furthermore, malonate uptake into the heart increased with duration of ischemia, suggesting that prior exposure to ischemic conditions cumulatively enhances malonate uptake upon subsequent reperfusion. This was confirmed *in vivo,* as DSM was only protective when administered at reperfusion and not when given prior to the onset of ischemia. Finally, we showed that malonate entry into the heart upon reperfusion is selective for the ischemic area at risk due to the low pH and is mediated by the monocarboxylate transporter 1 (MCT1). This novel mechanism of targeting ischemic tissue upon reperfusion makes DSM an attractive option for the clinical treatment of IR injury.

## Methods

Detailed methods and Major Resources Table can be found in the Data Supplement. Data will be made available upon reasonable request, by contacting a corresponding author.

## Results

### Malonate is protective in an MI model and is taken up selectively into the ischemic region of the heart

DSM is protective against cardiac IR injury, but its mechanism of protection remains uncertain. To address this gap in our knowledge we first tested the therapeutic range of DSM in IR injury. We infused DSM at a range of concentrations (1.6-160 mg/kg equivalent to ~11-1100 μmol/kg) at reperfusion in an *in vivo* murine LAD ligation model of MI. DSM infusion during this clinically-relevant reperfusion period led to a dose-dependent decrease in infarct size (Figure 1C), with 160 and 16 mg/kg showing robust cardioprotection. However, when DSM was infused prior to the onset of ischemia it was not protective (Figure 1D), even at 160 mg/kg which gave the smallest infarct when administered at reperfusion. This contrasted with the cell permeable dimethyl malonate (DMM), which can diffuse into the tissue and generate malonate, preventing succinate accumulation and thereby reducing IR injury.^18,25^ This suggests that either DSM is minimally taken up by tissues during normoxia and is only taken up by tissues after ischemia, or that DSM protects against acute IR injury by an extracellular mechanism.

We next measured malonate uptake into the ischemic and healthy heart tissue upon reperfusion in the LAD MI model. This showed that malonate was indeed taken up into cells within the infarct region to a level of ~400 pmol/mg tissue and to a far greater extent than into cells in the healthy tissue (Figure 1E). Furthermore, reperfusing with malonate slowed succinate oxidation within the infarct region with succinate remaining significantly elevated in the infarct region following 1 min reperfusion, compared to control reperfusion with saline (Figure 1F). Limiting succinate oxidation at reperfusion with malonate also blunted ROS production in the at-risk tissue (Figure 1G). We next assessed how SDH inhibition compared to direct inhibition of the mitochondrial permeability transition pore (PTP) with cyclosporin A (CsA). Here we found that the cardioprotection from malonate was additive to cyclosporin A alone (Figure 1H), hence targeting the upstream mechanism of PTP opening is an increasingly attractive option for IR injury treatment.

**Figure 2:**
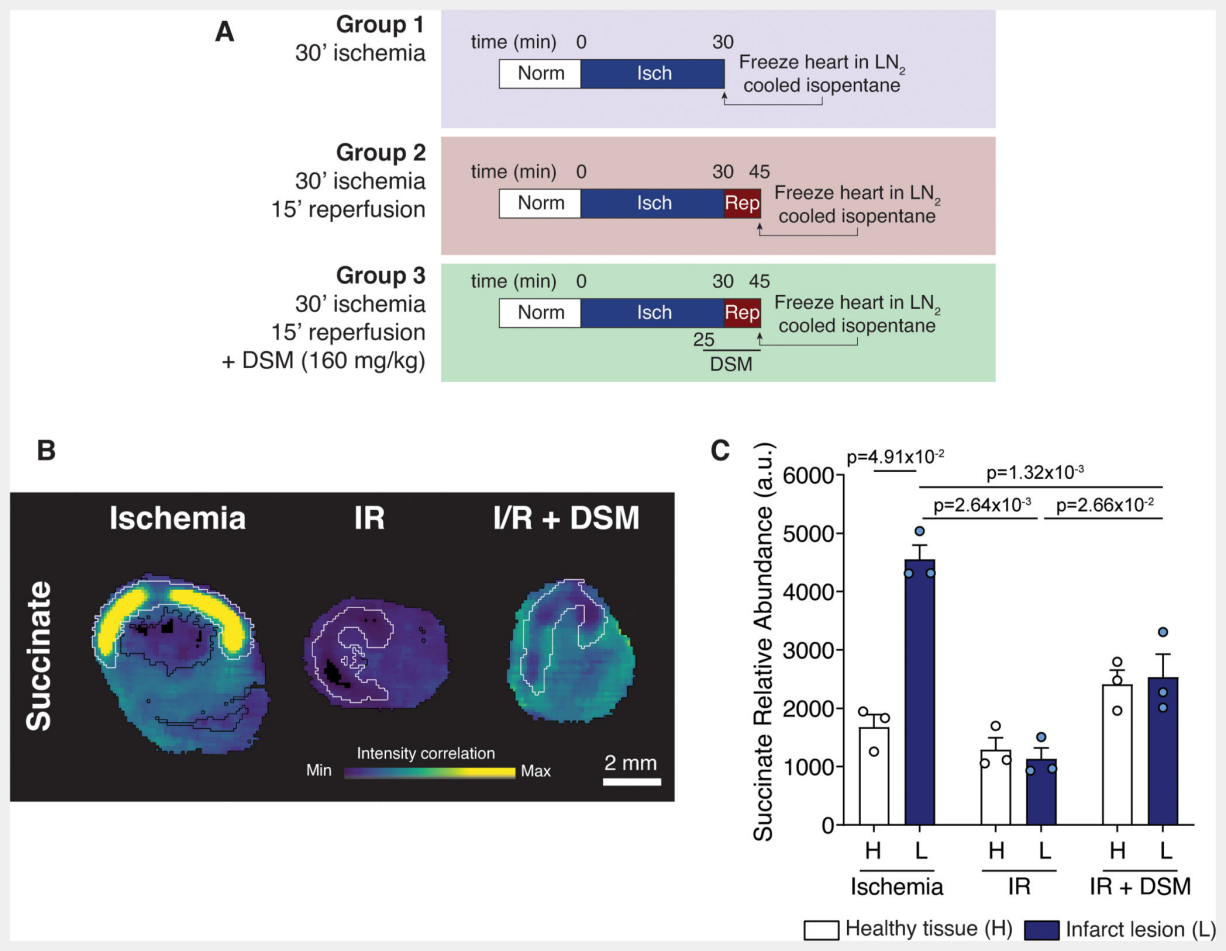
Localisation of succinate accumulation in heart tissue identified by mass spectrometry imaging. **A**, Outline of experimental groups for mass spectrometry imaging. **B**, Representative images of succinate abundance detected by mass spectrometry imaging in myocardial sections. Black outer line indicates the edge of the tissue slice, white inner line indicates infarct region. **C,** Quantification of succinate (**C**) identified by MSI (mean ± S.E.M, n=3 biological replicates, statistics: Friedman’s paired test for healthy vs lesion and Kruskal-Wallis with Dunn’s post hoc test between conditions).

To better understand the metabolic differences between the healthy and infarcted tissue upon malonate treatment, we investigated the *in vivo* MI model using mass spectrometry imaging (MSI). To do this we subjected hearts to either 30 min ischemia, 30 min ischemia before reperfusing the heart for 15 min or 30 min ischemia before reperfusing with DSM for 15 min (to mimic the cardioprotective malonate infusion) prior to snap freezing and processing for MSI (Figure 2A). Infarct lesions were demarcated using H&E, silver infarct staining and metabolite principal component analysis to differentiate the risk and non-risk regions. MSI coupled with the demarcated risk areas showed striking changes in the levels of succinate in the infarct region. During ischemia, succinate was significantly elevated in the at-risk tissue, with considerable succinate accumulation in the core of the infarct (Figure 2B and C). After 15 min reperfusion, the succinate levels in the infarct lesion returned to healthy tissue levels, due to succinate oxidation and efflux.^21^ When malonate was infused at the time of reperfusion, succinate levels remained higher in all regions of the heart than in the non-malonate treated heart, consistent with the prevention of its oxidation by SDH inhibition by malonate.

We conclude that malonate is indeed taken up by the heart, but this is greatly enhanced intoin ischemic region upon reperfusion. Therefore, malonate provides protection against cardiac IR injury *in vivo* over a range of concentrations following its selective entry into the infarct region, where it slows the oxidation of succinate during reperfusion, preventing the production of RET-derived ROS.

### Malonate Uptake into Cells is Inefficient at pH 7.4

To explore the mechanism of malonate uptake *in vitro* we incubated C2C12 and H9c2 myoblasts with DSM and measured intracellular malonate levels by LC-MS/MS. Incubation with DSM for 15 min at pH 7.4, led to a dose-dependent increase in intracellular malonate (Supplementary Figure 1A and D). Intracellular succinate also accumulated in a malonate-dependent manner (Supplementary Figure 1B and E), confirming that once malonate enters the cell it is rapidly transported into mitochondria and inhibits SDH. However, incubating cells with 1 mM DSM led to variable malonate uptake and little SDH inhibition, indicating that malonate uptake across biological membranes is inefficient. Moreover, the intracellular levels of malonate achieved by 250 μM of the malonate ester prodrug diacetoxymethyl malonate (MAM), are 80-fold more than that generated by incubation with 5 mM DSM.^25^ As DSM was protective in the LAD model when infused at 16 mg/kg, corresponding to a maximum possible blood malonate concentration of ~1.8 mM (assuming a blood volume of 1.5 ml in a 25 g mouse),^29^ this compares to the dose range tested in cells (furthermore, far more malonate is available for uptake *in vitro* than *in vivo* due to the large reservoir in the incubation medium). Even so, the intracellular malonate levels were far lower with DSM compared to MAM.^25^

The kinetics of cell uptake upon incubation with 5 mM DSM showed time dependent uptake, although extended incubation times were required for succinate elevation (Supplementary Figure 1C and F). This is likely due to the initial concentrations of malonate entering the cell being insufficient to inhibit SDH. These data are consistent with the lack of protection by DSM delivered prior to ischemia *in vivo,* as well as its limited uptake into normoxic tissues. Together these suggest that exposure to ischemia may facilitate malonate entry into the heart.

### Malonate Uptake into Cells and the Heart Can be Modulated by pH

**Figure 3:**
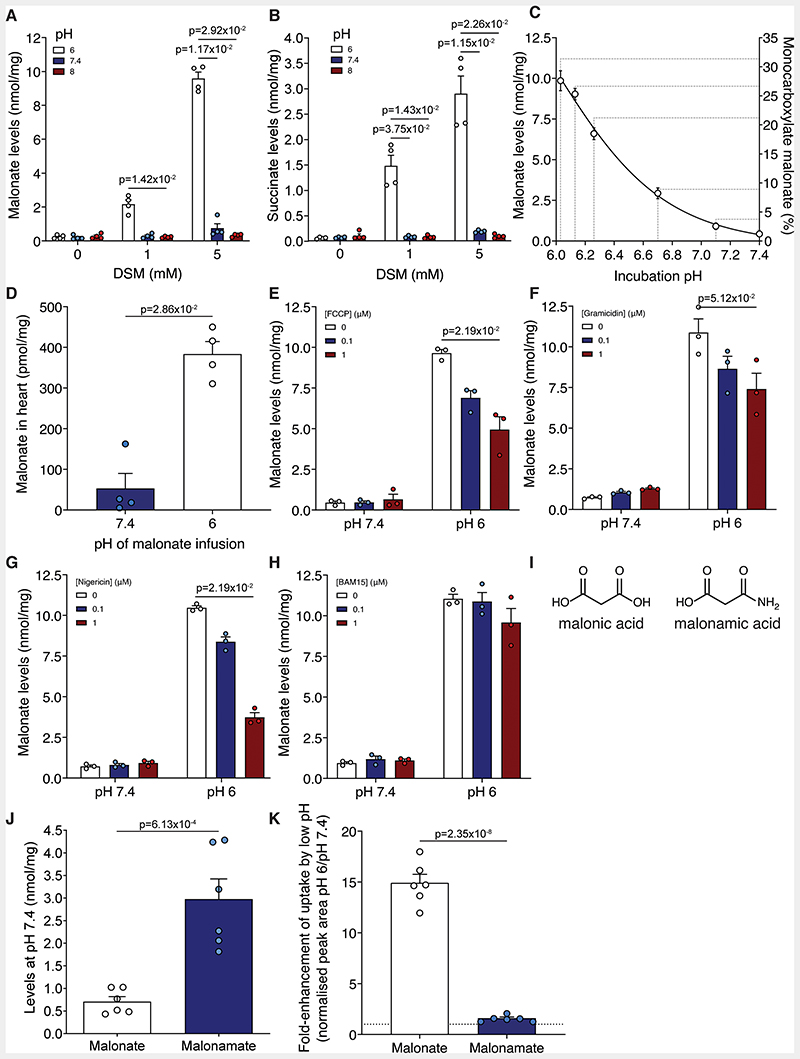
Malonate uptake is enhanced at low pH. **A and B**, C2C12 cells were incubated with DSM (0, 1 or 5 mM) for 15 min at either pH 6, 7.4 or 8 before measuring intracellular malonate (**A**) and succinate (**B**) by LC-MS/MS (mean ± S.E.M, n=4 biological replicates, statistics: Kruskal-Wallis with Dunn’s post hoc test). **C**, Malonate levels in C2C12 cells after incubation with DSM (5 mM) for 15 min at various (patho)physiological pH (mean ± S.E.M, n=3 biological replicates). **D**, Malonate levels in murine isolated Langendorff-perfused hearts treated with 5 mM DSM infused at either pH 7.4 or 6 for 5 min (mean ± S.E.M, n=4 biological replicates, statistics: unpaired, two-tailed Mann-Whitney U test). **E** to **H**, C2C12 cells were incubated with DSM (5 mM) for 15 min at either pH 6 or 7.4 in the presence of FCCP (**E**), gramicidin (**F**), nigericin (**G**) or BAM15 (**H**) before measuring intracellular malonate by LC-MS/MS (mean ± S.E.M, n=3 biological replicates, statistics: Kruskal-Wallis with Dunn’s post hoc test). **I**, Structures of malonic acid and malonamic acid. **J**, Quantification of levels of malonate and malonamate in C2C12 cells after incubation (5 mM DSM or disodium malonamate; 15 min) at pH 7.4 (n=6 biological replicates). **K**, C2C12 cells were incubated with either DSM or disodium malonamate (both 5 mM) at pH 6 or 7.4 for 15 min and the intracellular levels measured by LC-MS/MS. (mean ± S.E.M. of the fold-enhancement of uptake at pH 6 versus pH 7.4, n=6 biological replicates). Statistics for **J** and **K**: unpaired, two-tailed Student’s t-test test).

Malonate is a dicarboxylate at physiological pH (Figure 1A, pKa = 2.83 and 5.69), suggesting that the pH decrease in ischemic tissue^9,11,30^ may enhance malonate uptake into the heart during early reperfusion, by increasing the concentration of its monocarboxylic form. Incubating cells with DSM at either pH 6, 7.4 or 8 for 15 min, led to large differences in malonate uptake (Figure 3A and Supplementary Figure 2A). At pH 6, the levels of malonate in the cell were significantly higher than at pH 7.4 or 8, thus malonate uptake is favoured by acidic pH. Succinate levels mirrored those of malonate, with greater succinate accumulation as a result of increased malonate-dependent SDH inhibition at low pH (Figure 3B and Supplementary Figure 2B). Low pH alone had no effect on succinate levels (Figure 3B and Supplementary Figure 2B), suggesting that it was due to malonate entry into the cells followed by SDH inhibition.

Incubating cells with malonate over a range of pH values between pH 7.4 and 6 clearly showed the pH-dependent uptake of malonate, in line with the increase in monocarboxylate malonate proportion (Figure 3C). This was mirrored by succinate levels (Supplementary Figure 2C). Therefore, even a small drop in pH can lead to a substantial increase in malonate uptake, and thus is likely to be relevant for its entry into ischemic tissue upon reperfusion.

As the cultured cells used are non-contractile and substantially differ in their properties from *in situ* contracting cardiomyocytes,^15^ importantly, we next assessed whether decreasing the pH also enhanced malonate uptake into the *ex vivo* perfused Langendorff heart. Malonate was infused into the heart at either pH 7.4 or 6 for 5 min, before briefly flushing at pH 7.4 to remove non-myocardial malonate. Remarkably, the levels of malonate were significantly elevated when infused at pH 6 compared to 7.4, confirming that the *in vitro* results translate to the heart (Figure 3D). We conclude that low pH conditions facilitate the entry of malonate into cardiomyocytes.

### Malonate Uptake Can be Perturbed by Modulating the Plasma Membrane H^+^ Gradient

As pH modulated malonate entry into cells *in vitro* and in the heart, we next probed the factors affecting malonate uptake into cells. Malonate entry into cells at either pH 7.4 or 6 was blocked at 4 °C and was associated with negligible succinate levels (Supplementary Figure 2D and E). That a decrease in extracellular pH increased malonate entry into cells, suggested uptake driven by the proton gradient. Therefore, we next abolished the plasma membrane proton gradient using ionophores^21^ which prevented the cellular uptake of malonate (Figure 3E to G and Supplementary Figure 3A) and subsequent increase in succinate levels (Supplementary Figure 3B to E). However, the uncoupler BAM15, which is selective for the mitochondrial inner membrane over the plasma membrane,^31^ had little effect, consistent with the plasma membrane proton gradient driving malonate uptake (Figure 3H and Supplementary Figure 3F).

We next used non-specific transport inhibitors to assess whether we could block malonate uptake at low pH. DIDS, an irreversible inhibitor of chloride/bicarbonate exchange that inhibits malonate uptake into erythrocytes,^32,33^ led to a dose-dependent inhibition of malonate uptake, although succinate remained high (Supplementary Figure 3G and H). Thus, malonate uptake into cardiomyocytes at low pH is driven by a proton gradient through a transporter-dependent process.

Malonate’s pKa’s are 2.83 and 5.69, so at pH 6.4, approximately 16% of the malonate would be in its monocarboxylate form. To determine whether the uptake of malonate was dependent on its protonation to a monocarboxylate form, we assessed the uptake of a compound that mimics the monocarboxylate malonate. In 3-amino-3-oxopropionate (malonamate; Figure 3I), one carboxylic acid has been replaced with a neutral amido group leaving a single carboxylic acid of pKa ~4.75. Thus, at pH 7.4 malonamate resembles the monocarboxylate form of malonate. This led to more of malonamate being taken up into cells at pH 7.4 than malonate (Figure 3J). Lowering the pH to 6 led to an ~15-fold increase in malonate uptake while malonamate uptake changed negligibly (Figure 3K), because it will remain as a monocarboxylate across this pH range.

Together, these data support transport of the monoanionic form of malonate, under the conditions that occur during early reperfusion. Therefore, a pH gradient can drive the uptake of malonate via protonation and transport in its monocarboxylate form.

### Malonate Uptake Under Reperfusion Conditions is Dependent on MCT1

**Figure 4:**
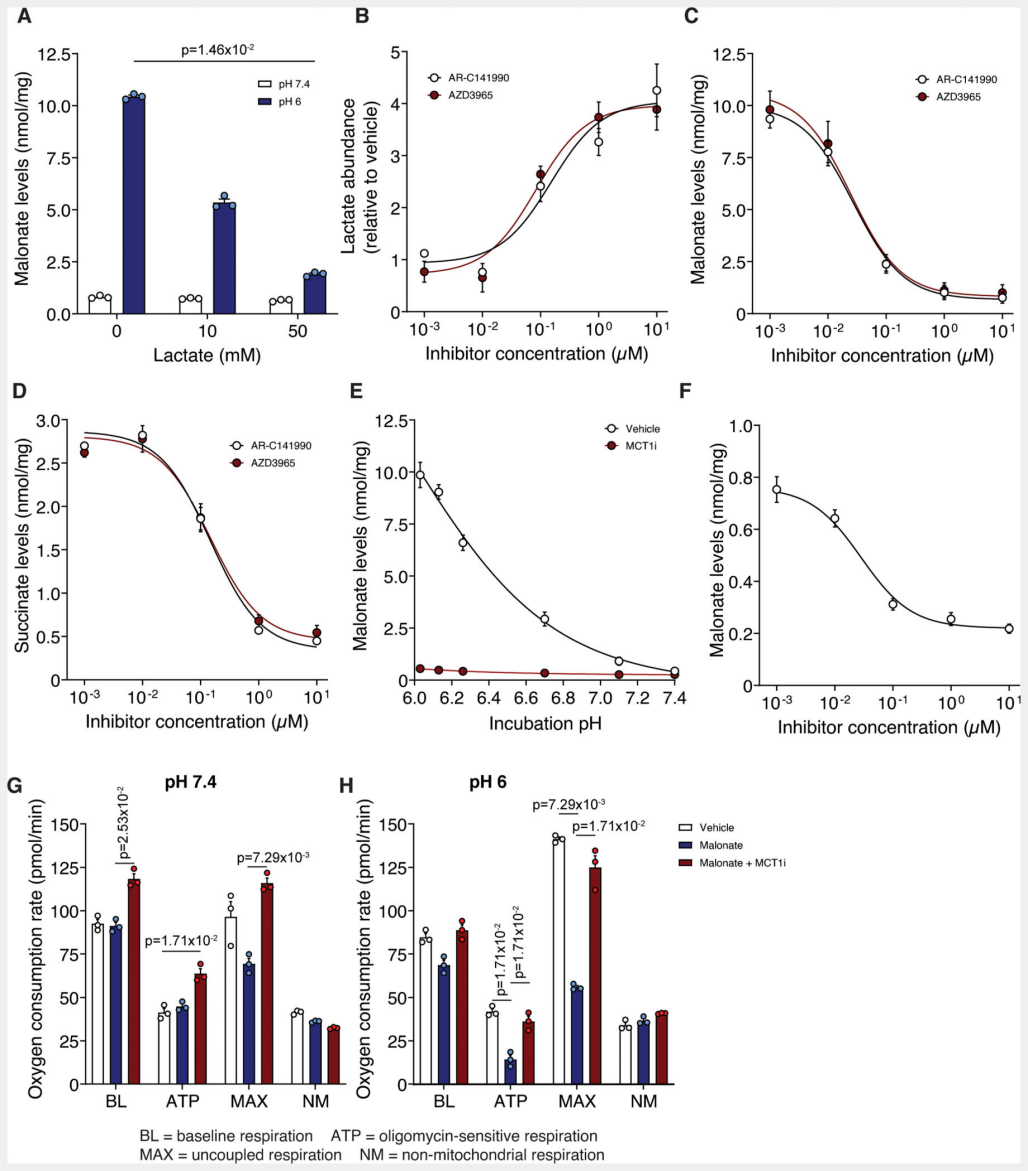
Inhibition of MCT1 prevents the enhanced uptake of malonate at lowered pH. **A**, Malonate uptake (5 mM DSM, 15 min) in C2C12 cells in the presence of lactate (0, 10 or 50 mM). **B**, Lactate levels in C2C12 cells after 15 min treatment with varying concentrations of MCT1 inhibitors. **C** and **D**, Effect of MCT1 inhibition by AR-C141990 or AZD3965 on malonate (5 mM DSM, 15 min) uptake (**C**) at pH 6 and subsequent succinate levels (**D**). **E**, C2C12 cells were incubated with DSM (5 mM) for 15 min at various pH ± 10 μM AR-C141990. **F**, MCT1 inhibition by AR-C141990 on malonate (5 mM DSM, 15 min) uptake at pH 7.4 (**A** to **F**, mean ± S.E.M., n=3 biological replicates, statistics: (**A**) Kruskal-Wallis with Dunn’s post hoc test). **G** and **H**, Effect of malonate (5 mM DSM) on cellular oxygen consumption at pH 7.4 (**G**) or 6 (**H**) ± MCT1 inhibitor (10 μM AR-C141990) (data presented as non-mitochondrial respiration normalized mean oxygen consumption rate (OCR) ± S.E.M of 3 biological replicates (n=12-16 technical replicates per ‘biological replicate’), statistics: Kruskal-Wallis with Dunn’s post hoc test). BL = baseline OCR, ATP = OCR in the presence of 1.5 μM oligomycin, MAX = OCR in the presence of 1 μM FCCP, NM = OCR in the presence of 4 μg/ml rotenone and 10 μM antimycin A.

The inhibitory effect of DIDS, which also inhibits MCT1,^34^ raised the possibility of the uptake of the protonated form of malonate being catalysed by a monocarboxylate transporter (MCT; SLC16). MCT1 (SLC16A1) is the principal transporter of lactate, which is carried in symport with a proton, both of which are elevated during ischemia, making MCT1 an attractive candidate transporter for malonate uptake upon reperfusion. Additionally, it was recently shown that MCT1 mediates the efflux of succinate from the ischemic heart during reperfusion and from exercising muscle.^21,35^ As MCT1 is a lactate transporter, we first assessed whether high concentrations of lactate in the incubation medium compete with malonate. Excess lactate led to a concentration-dependent decrease in malonate uptake into the cell, and a corresponding decrease in succinate accumulation (Figure 4A and Supplementary Figure 4A). This suggested that at a low pH, malonate uptake into the cells is via MCT1, which is highly expressed in cardiomyocytes.^36^

To confirm that MCT1 was the malonate transporter, we assessed the potent and selective MCT1 inhibitors, AR-C141990 and AZD3965.^21,35,37,38^ When cells were incubated with these MCT1 inhibitors, they led to a dramatic dose-dependent increase in lactate levels within the cell, consistent with preventing lactate efflux via MCT1 (Figure 4B). MCT1 inhibition by either AR-C141990 or AZD3965 led to a profound dose-dependent decrease in malonate uptake (Figure 4C). Inhibition of malonate uptake by MCT1 inhibition led to a corresponding decrease in succinate accumulation within cells (Figure 4D). Additionally, a time course of malonate uptake at low pH, showed that MCT1 inhibition largely abolished the increase in malonate over time, along with the corresponding increase in succinate (Supplementary Figure 4B and C).

MCT1 inhibition blocked the pH-dependent increase in malonate uptake (Figure 4E) and in parallel prevented succinate accumulation (Supplementary Figure 4D). The MCT1 inhibitor led to a dose-dependent inhibition of malonate uptake even at pH 7.4 (Figure 4F). Therefore, malonate can be transported by MCT1 at pH 7.4, but this is greatly enhanced at lower pH due to the increased proportion of malonate in its monocarboxylate form.

We next assessed the impact of the malonate taken up into cells on mitochondrial function by measuring respiration. Lowering pH itself had little effect; however, addition of malonate severely reduced cellular respiration (Figure 4G and H and Supplementary Figure 4E and F) and this effect was rescued by MCT1 inhibition (Figure 4G and H and Supplementary Figure 4E and F). MCT1 inhibition led to a small increase in oxygen consumption at baseline, which may be due to lactate accumulation increasing the NADH/NAD+ ratio driving mitochondrial respiration.^39^

**Figure 5:**
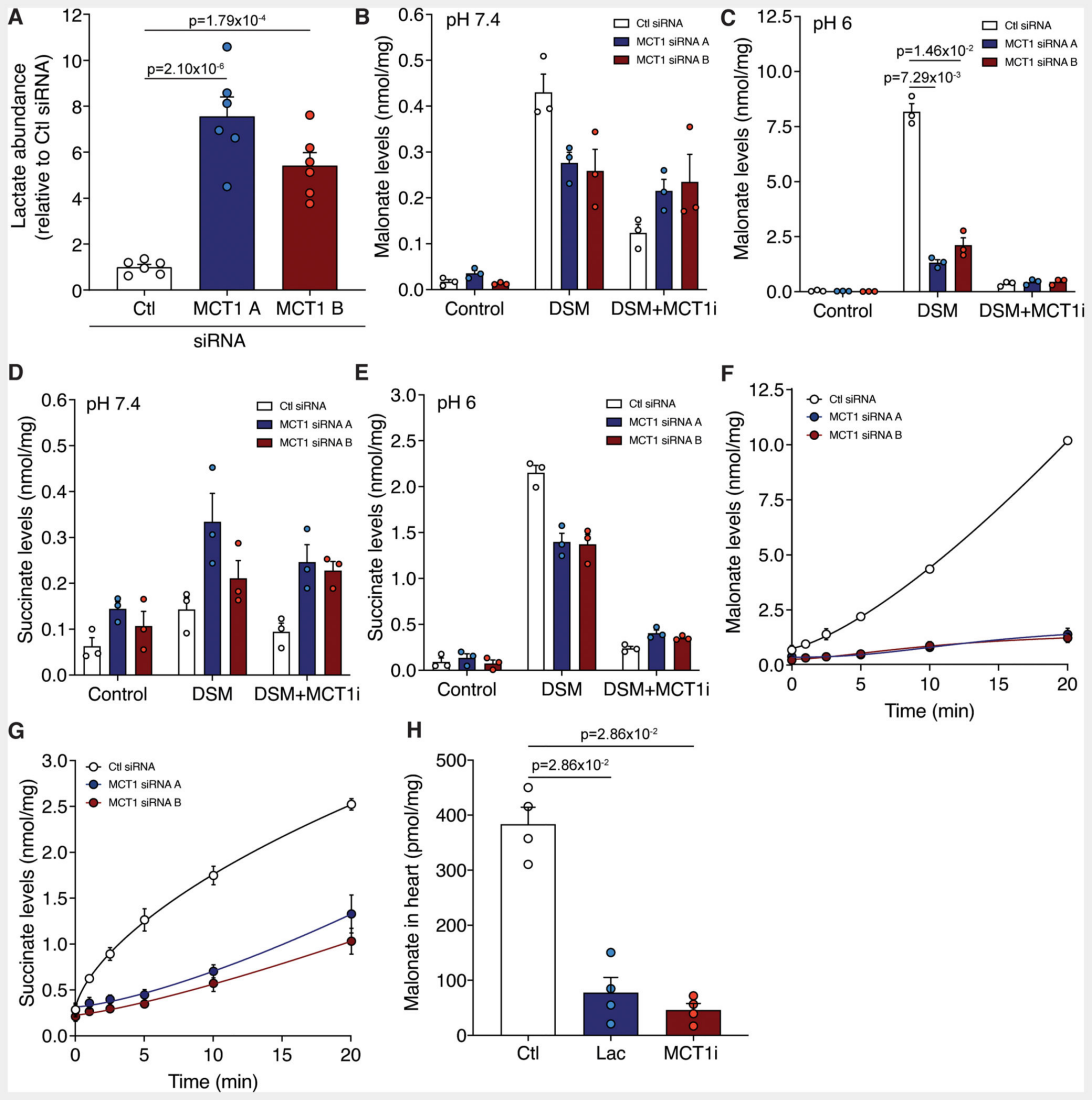
Genetic knockdown of MCT1 prevents the uptake of malonate. **A**, Relative lactate levels in C2C12 cells treated with control or MCT1 siRNA (mean ± S.E.M. of lactate levels relative to control, n=6 biological replicates, statistics: one way-ANOVA with Bonferroni’s post hoc test). **B** to **E**, Incubation of malonate (5 mM DSM) in MCT1 KD cells at pH 7.4 (**B** and **D**) or 6 (**C** and **E**) for 15 min ± MCT1i (10 μM AR-C141990). Levels of malonate (**B** and **C**) and succinate (**D** and **E**) quantified by LC-MS/MS (mean ± S.E.M., n=3 biological replicates, statistics: Kruskal-Wallis with Dunn’s post hoc test). **F** and **G**, Time course of malonate uptake (5 mM DSM) at pH 6 in MCT1 KD cells (**F**) and corresponding succinate levels (**G**) (mean ± S.E.M., n=3 biological replicates). **H**, Malonate levels in murine Langendorff hearts perfused at pH 6 for 5 min with 5 mM DSM ± lactate (50 mM; Lac) or MCT1 inhibitor (10 μM AR-C141990; MCT1i) (mean ± S.E.M., n=4, statistics: unpaired, two-tailed Mann-Whitney U test versus control).

To confirm the pharmacological effects of MCT1 inhibition on malonate uptake genetically, we knocked down (KD) MCT1 using siRNA in both HeLa and C2C12 cells (Supplementary Figure 5A to D). Consequently, lactate levels in MCT1 KD cells were significantly elevated compared to the control siRNA (Figure 5A). When MCT1 KD cells were incubated with malonate at low pH, malonate uptake was dramatically blocked (Figure 5B to E and Supplementary Figure 5C), confirming MCT1 is directly responsible for malonate transport at low pH. Residual malonate uptake in the MCT1 KD cells was inhibited by cotreatment with an MCT1 inhibitor, further confirming its MCT1-dependence (Figure 5F and Supplementary Figure 5F). Additionally, succinate was elevated in the MCT1 KD cells compared with control siRNA cells, a result not seen with acute pharmacological MCT1 inhibition (Figure 5D). This suggests that MCT1 may also play an important role in normoxic succinate metabolism and homeostasis and not just during myocardial reperfusion and intense exercise.^21,35^ Intriguingly, the succinate levels after malonate treatment over time differed between the two cell types, with succinate levels remaining low in C2C12 KD but elevated in HeLa MCT1 KDs compared to controls, suggesting a reliance on MCT1 for succinate efflux (Figure 5G and Supplementary Figure 5G).

As HeLa cells constitutively express MCT4, which is under the control of hypoxia inducible factor (HIF-1α) and may be implicated in the transport of metabolites in IR, we knocked down MCT4 independently of MCT1 (Supplementary Figure 5D and E). MCT4 KD had little effect on malonate uptake, with malonate and succinate levels mirroring those of control siRNA treated cells (Supplementary Figure 5F and G). Thus, MCT1 is the main driver of malonate uptake at lowered pH.

Overall, diminishing MCT1 activity, either by pharmacological inhibition or genetic knockdown, impairs malonate uptake at both acidic and ‘normal’ physiological pH. In addition, this is the first evidence that MCT1 can transport a 3-carbon chain length dicarboxylate, which may also have implications in normal physiology and other pathologies.

Finally, we assessed if MCT1 was responsible for malonate uptake at lowered pH in the heart. Malonate uptake into the Langendorff perfused heart was blocked by either excess lactate, or the MCT1 inhibitor AR-C141990, in the perfusion medium (Figure 5H). Together these findings confirm that MCT1 is responsible for the low pH uptake of malonate, both in cells and in the intact heart.

### Ischemic Conditions are Sufficient to Drive Malonate Uptake into the Heart Upon Reperfusion

**Figure 6:**
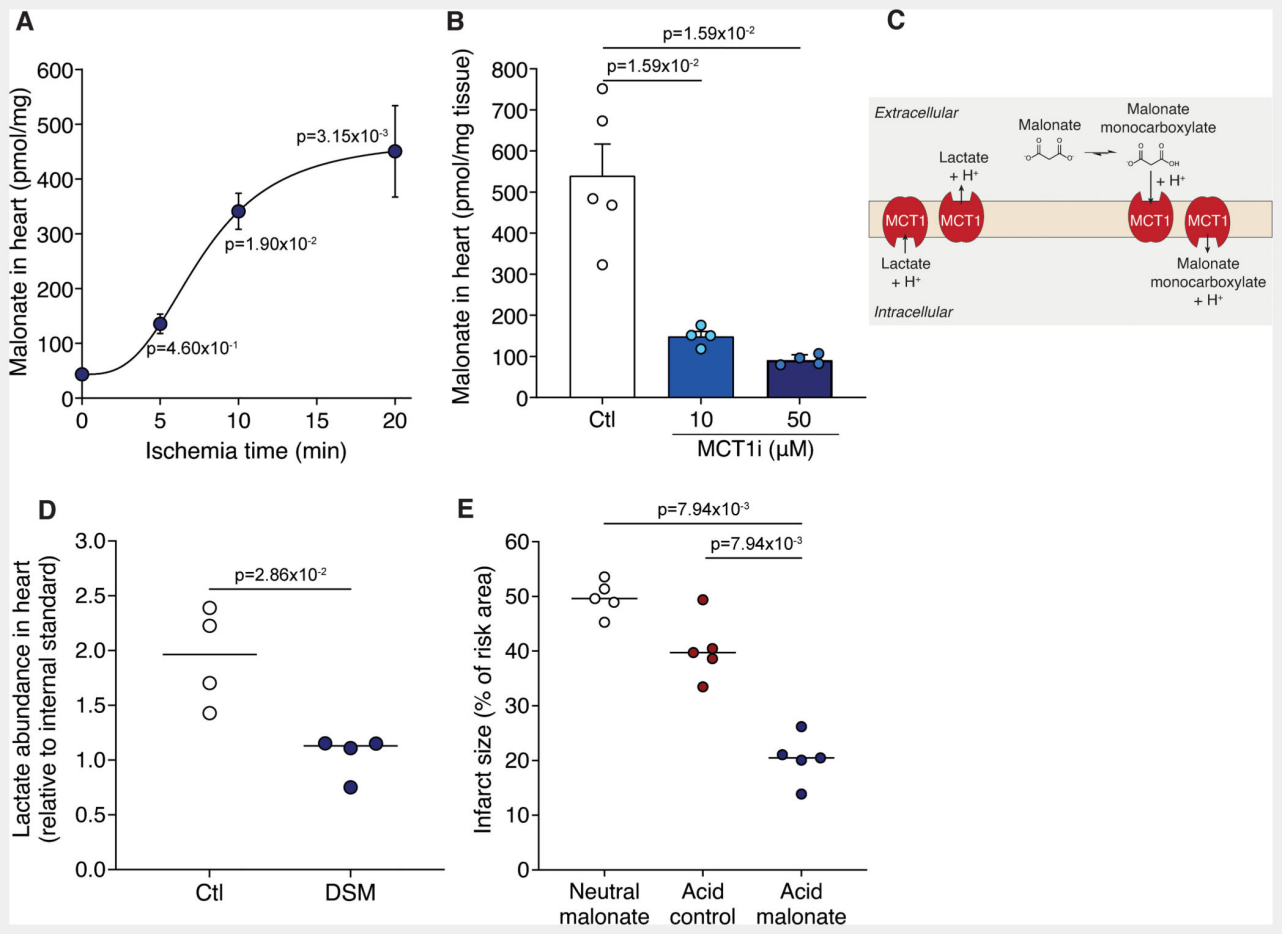
Ischemia drives malonate protonation, uptake and cardioprotection. **A**, Langendorff-perfused murine hearts were held ischemic for either 0, 5, 10 or 20 min and reperfused with 5 mM DSM (pH 7.4) before malonate levels measured in the heart by LC-MS/MS (mean ± S.E.M, n=4 (control, 5 min) or 6 (10 and 20 min) biological replicates, statistics: Kruskal-Wallis with Dunn’s post hoc test). **B**, Malonate levels in murine Langendorff hearts exposed to 20 min ischemia and reperfused with 5 mM malonate (pH 7.4) ± MCT1 inhibitor (10 or 50 μM AR-C141990) (mean ± S.E.M, n=4-5 biological replicates, statistical significance assessed by unpaired, two-tailed Mann-Whitney U test versus control). **C**, Model of potential lactate and malonate exchange during reperfusion. **D**, Lactate levels in the Langendorff heart after 20 min ischemia and 1 min reperfusion ± 5 mM DSM (mean, n=4 biological replicates, statistics: two-tailed, unpaired Mann-Whitney U test). **E**, Infarct size in murine LAD MI model with 100 μl bolus of 8 mg/kg DSM, pH 4 acid control or 8 mg/kg pH 4 formulated malonate at reperfusion after 30 min ischemia (mean, n=5 biological replicates, statistics: two-tailed, unpaired Mann-Whitney U test vs acid malonate).

That low pH enhanced malonate entry into heart cells was consistent with the protection afforded by DSM in IR injury being due to the local decrease in pH during ischemia and initial reperfusion. To test this hypothesis, Langendorff perfused hearts were held ischemic for various times before DSM was infused for 5 min, followed by flushing, and measurement of malonate. The malonate levels in heart tissue were dependent on ischemia-time, with the highest occurring after 20 min ischemia and being ~10-fold greater than into the normoxic heart (Figure 6A). This malonate uptake into the ischemic Langendorff perfused heart was dramatically reduced by the MCT1 inhibitor AR-C141990 (Figure 6B). This confirmed that malonate uptake into the ischemic heart upon reperfusion is a selective process, driven by the low pH and facilitated by the MCT1.

During ischemia, the pH of ischemic tissue lowers but in addition, lactate accumulates and upon reperfusion can be transported out of cardiomyocytes by MCT1. This could enhance malonate uptake, as the MCT1 may then act as a lactate-H^+^/monocarboxylate malonate-H^+^ exchanger (Figure 6C). To assess this, we measured lactate levels in malonate-perfused IR Langendorff hearts. We found that compared to reperfusion alone, lactate levels decreased in the malonate-treated hearts (Figure 6D). Furthermore, malonate treatment decreased lactate levels in cells that had been treated with the mitochondrial inhibitor antimycin A to enhance lactate production (Supplementary Figure 6A). This suggests that extracellular malonate and a proton exchange for intracellular lactate, helping drive malonate entry into cardiomyocytes upon reperfusion and contributing to its at-risk tissue-selective effect.

Therefore, malonate uptake into the heart upon reperfusion is dependent on the tissue having first undergone a period of ischemia, leading to a drop in pH and an accumulation of lactate.

### Low pH Formulation Improves Cardioprotection by Malonate

The mechanism of malonate uptake into cardiac tissue suggested that lowering the pH of the malonate infusion would increase the proportion in the monocarboxylate form and thereby increase its potency as a cardioprotective agent. To assess this, we used a malonate dose which was not protective (8 mg/kg) when administered at a neutral pH in the *in vivo* LAD MI model. When this dose of malonate was reformulated at pH 4 (a pH currently used in FDA-approved parenteral formulations)^40^ and administered as a bolus, this conferred significant protection that was not due to the low pH alone (Figure 6E and Supplementary Figure 6B). Furthermore, while neutral malonate administered before ischemia was not protective, acidified malonate drove its uptake into cardiomyocytes and significantly reduced infarct size when infused before ischemia (Supplementary Figure 6C in the Data Supplement).

## Discussion

No medicine is currently available that can be given at reperfusion to prevent cardiac IR injury.^1,6^ Drugs that prevent cardiac IR injury should both reduce MI damage and the subsequent development of HF.^1,12^ Targeting succinate metabolism has been shown to be a promising therapeutic approach. Inhibiting SDH using the reversible, competitive inhibitor malonate reduces infarct size in small and large animal models of cardiac IR injury, despite its mechanism of entry into heart tissue being unknown.^26,27^

Here, we found that malonate uptake into cells and the heart at pH 7.4 was inefficient. However, the uptake of malonate into cells was dramatically enhanced at the lower levels of pH that occurs during ischemia and by lactate accumulation within cells. Thus, ischemia provides an environment that will protonate malonate and thereby enable its uptake by the MCT1 transporter. Interestingly, this transporter undergoes a *trans*-acceleration phenomenon, whereby its transition from the outward-facing to the inward-facing conformation occurs more rapidly in the presence of a *trans* substrate.^41,42^ In this case, the uptake of malonate on the extracellular side of the plasma membrane may be accelerated by lactate efflux. In the LAD MI model of IR injury, malonate is readily available at the point of reperfusion, thus a proportion of the malonate would be protonated and accessible for transport by MCT1. This enables malonate entry into the heart in an at-risk tissue-selective manner.

Additionally, as well as the inhibition of SDH, accelerating lactate efflux from the heart may also play a role in the reduction of IR injury. By shifting the equilibrium to facilitate anaerobic ATP production this may promote the early restoration of ionic gradients through ATP-dependent transporters.^43,44^ Furthermore, facilitating lactate efflux may promote the extrusion of protons from the intracellular environment, thus reducing the activity of H^+^ transporters such as the Na^+^-H^+^ exchanger, though further investigation into these mechanisms is warranted.^43,45^

This is the first account of an ischemia-selective cardioprotective agent, utilising the pathological differences between risk and non-risk tissue to drive uptake. As MCT1 is highly expressed in the hearts of mice, rats, pigs and humans (Supplementary Figure 6D) and the characteristics of ischemia are conserved between these species,^19,24^ these drivers for malonate uptake and the plasma membrane transporter may facilitate malonate cardioprotection in humans. In addition to its place in the treatment of IR injury in MI, the mechanism of ischemia-enhanced delivery of malonate may provide a novel treatment option for IR injury under many other circumstances.

There is much interest in targeted drug delivery and the ability to engage the intended site while reducing off-target effects. Here, we have shown that malonate is not only a cardioprotective agent, but it does this while its entry into the rest of the heart is limited. Thus, malonate is a potent and ischemic tissue-targeted drug, explaining how large doses can be delivered acutely with minimal toxic effects, which is likely to be important bearing in mind the many comorbidities associated with MI.^46^ Furthermore, as malonate can be efficiently metabolised^28,47^ and has the potential to promote cardiomyocyte regeneration^48^.

Malonate is robustly protective in acute IR injury, though further work is now required to understand the tractability of malonate treatment in chronic IR injury models; in particular, conducting a double-blind chronic large animal IR injury study.^49^ This would provide the greatest insight into the cardioprotection capabilities of malonate post MI and its effect on the development of heart failure and define its potential for translation to the clinic.

## Conclusions

We have shown that malonate is an ischemia-selective drug, due to the lowered pH and lactate accumulation of ischemic tissue driving its uptake via the MCT1. Furthermore, we show that a low pH formulation of malonate enhances its therapeutic potency. Malonate is the first at-risk tissue-selective cardioprotective drug and represents a significant step towards the treatment of IR injury.

## Supplementary Material

Graphical Abstract

Long In Vivo Checklist

Supplemental Material

Uncut Gel Blots

Major Resources Table

## Figures and Tables

**Figure 7:**
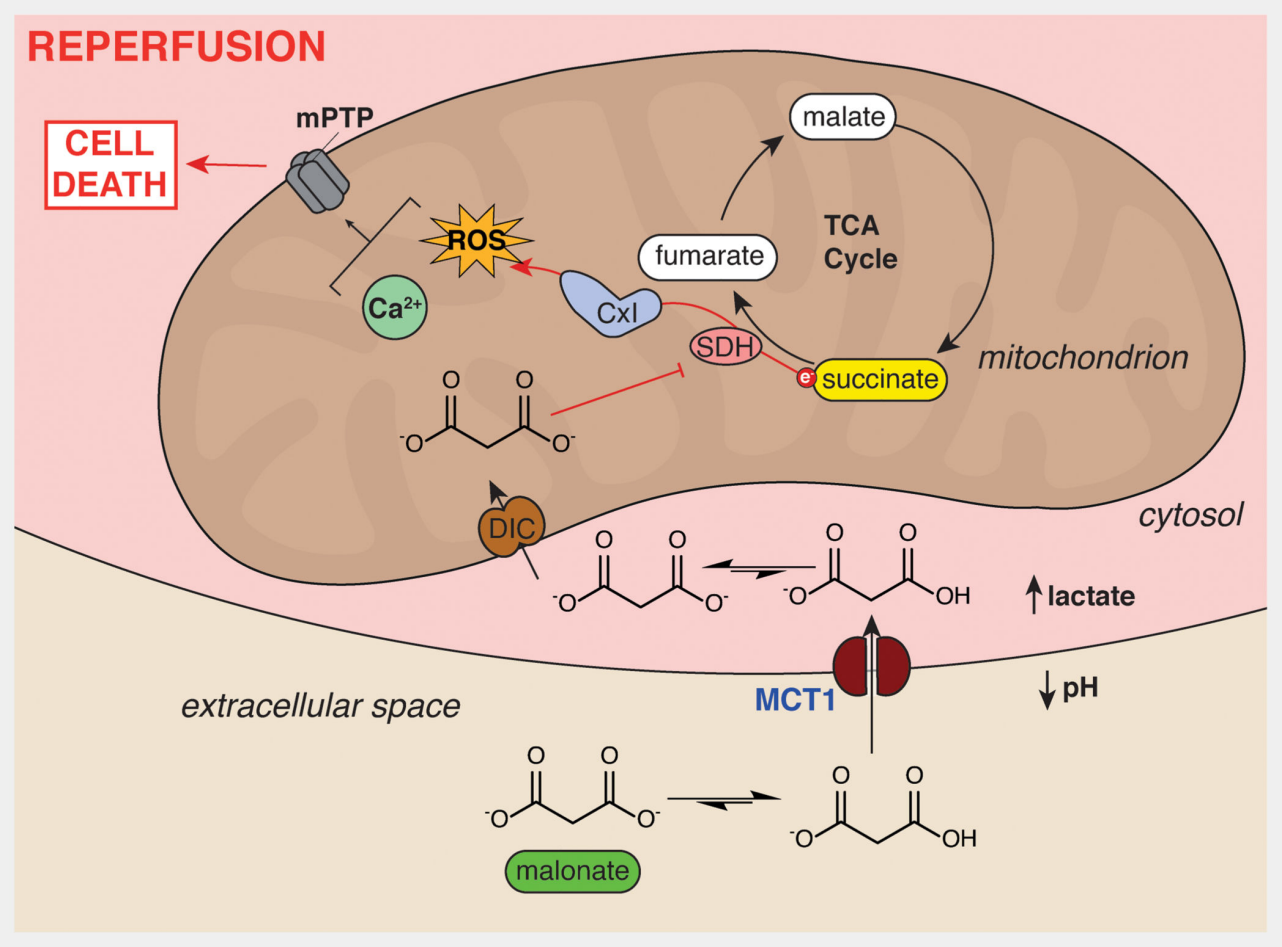
Schematic of ischemia-dependent malonate uptake via MCT1. The accumulation of lactate and protons in ischemic tissue and equilibration with the extracellular space facilitates protonation of malonate to its monocarboxylate form. This enables it to be an MCT1 substrate and enter cardiomyocytes upon reperfusion. Here, the malonate is transported into mitochondria by the mitochondrial dicarboxylate carrier where it can subsequently go on to inhibit SDH. SDH inhibition reduces succinate oxidation, ROS production by RET through complex I and opening of the mitochondrial permeability transition pore, thereby reducing cell death from IR injury. *CxI, complex I; DIC, mitochondrial dicarboxylate carrier (SLC25A10); mPTP, mitochondrial permeability transition pore*.

## Novelty and Significance

### What is known?

- Extensive succinate accumulation during ischemia and its subsequent rapid oxidation on reperfusion drives ischemia/reperfusion (IR) injury.
- Preventing succinate accumulation during ischemia reduces damage on reperfusion.
- Inhibiting succinate dehydrogenase with malonate is protective against IR injury though its mechanism of entry into cardiomyocytes is undefined.

## Non-Standard Abbreviations And Acronyms

RET: reverse electron transport
mPTP: mitochondrial permeability transition pore
DMM: dimethyl malonate
DSM: disodium malonate
MAM: diacetoxymethyl malonate
MSI: mass spectrometry imaging
CsA: Cyclosporin A
