# Supplementary figures and images for: Ischemia-Selective Cardioprotection by Malonate for Ischemia/Reperfusion Injury
Source: Circ Res. Author manuscript; Available in PMC 2022 Sep 9. (PMC9426742; doi:10.1161/CIRCRESAHA.121.320717)

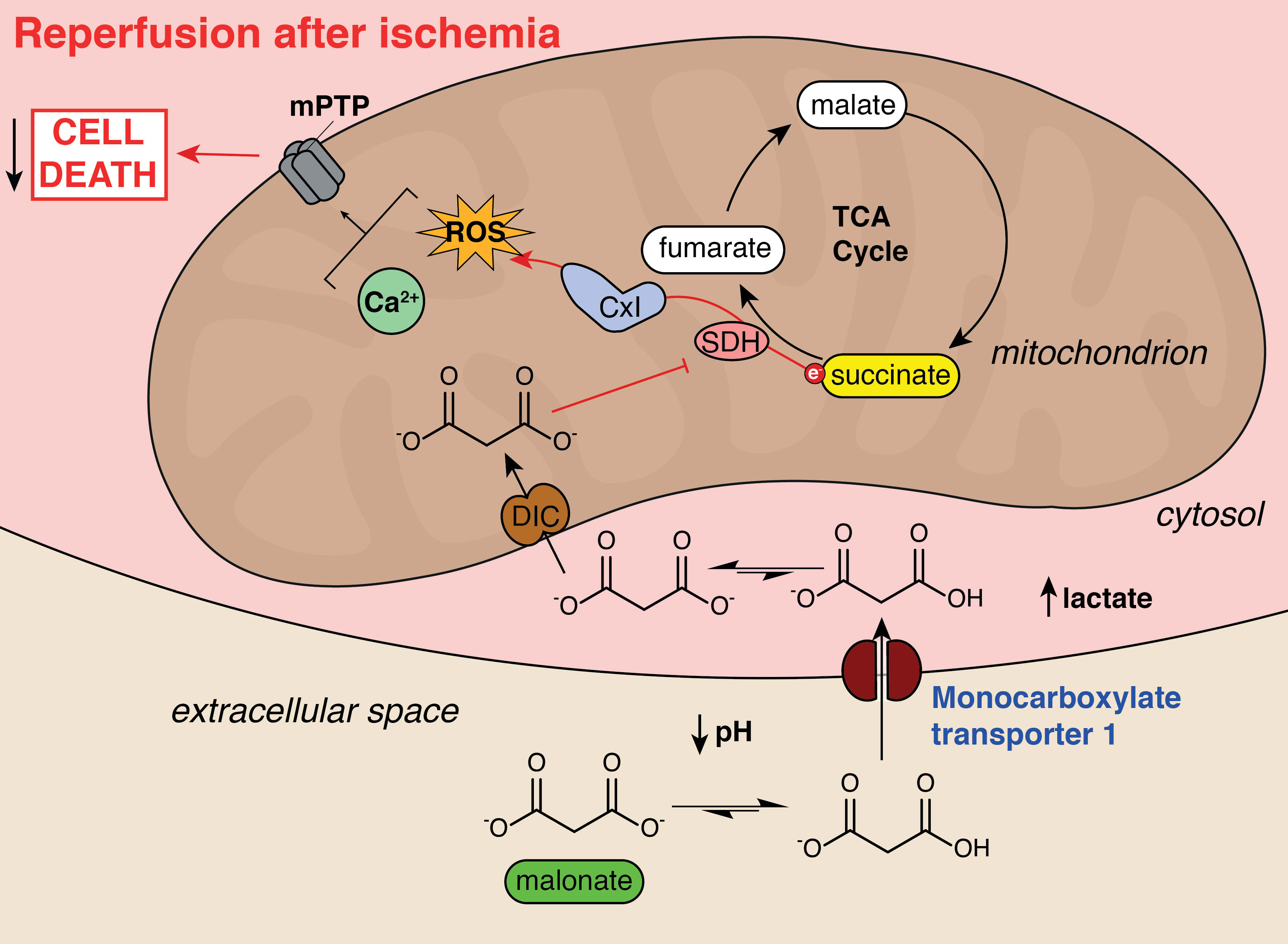

Supplement: Graphical Abstract [file EMS152108-supplement-Graphical_Abstract.jpg]
