# Supplementary material for: Ischemia-Selective Cardioprotection by Malonate for Ischemia/Reperfusion Injury
Source: Circ Res. Author manuscript; Available in PMC 2022 Sep 9. (PMC9426742; doi:10.1161/CIRCRESAHA.121.320717)
Supplement: Supplemental Material [file EMS152108-supplement-Supplemental_Material.pdf]

SUPPLEMENTAL MATERIAL

Ischemia-Selective Protection Against Cardiac Ischemia/Reperfusion Injury by Malonate

Hiran A. Prag, Dunja Aksentijevic, Andreas Dannhorn, Abigail V. Giles, John F. Mulvey, Olga Sauchanka, Luping Du, Georgina Bates, Johannes Reinhold, Duvaraka Kula-Alwar, Zhelong Xu, Luc Pellerin, Richard J. A. Goodwin, Michael P. Murphy* and Thomas Krieg*

*Correspondence to:

Thomas Krieg, MD, Department of Medicine, University of Cambridge, Cambridge, CB2 0QQ, UK. Email: tk382@medschl.cam.ac.uk

Michael P. Murphy, PhD, MRC Mitochondrial Biology Unit, University of Cambridge, Cambridge, CB2 0XY, UK. Email: mpm@mrc-mbu.cam.ac.uk

DETAILED METHODS

Animals

Procedures were carried out in accordance with the UK Animals (Scientific Procedures) Act of 1986 and the University of Cambridge Animal Welfare Policy under project licence 70/8238 and 70/7963, reviewed by the University of Cambridge Animal Welfare Ethical Review Board or the Tianjin Medical University Animal Care and Use Committee. All animal experiments described were carried out in accordance with the Guide for the Care and Use of Laboratory Animals, published by the US National Institutes of Health (NIH Publication No. 85-23, revised 1996). C57BL/6J mice (all male, 8-12 weeks) were ordered from Charles River Laboratories UK (Margate, UK) or the Institute of Laboratory Animal Science, Chinese Academy of Medical Sciences (Beijing, China). Both were maintained with ad libitum chow and water. Only male mice were used in the study as this provided the best comparison to previous work carried out.

Maintenance of Cells in Culture

C2C12 (mouse), H9c2 (rat) and HeLa (human) cells were obtained from American Type Culture Collection (ATCC). All were maintained at 37 °C, 5% CO_2 and 100% humidity. Cell media was changed every 2-3 days with passaging at <80% confluency and seeding at no higher than 5 x 10^3 viable cells/cm^2 with a typical subcultivation ratio of 1:5-1:10. Cell media was DMEM media (4.5 g/l glucose, 1 mM sodium pyruvate, 2 mM Glutamax, 1.5 g/l sodium bicarbonate; Gibco, UK)) with 10% Fetal Bovine Serum (FBS; Gibco, UK).

LC-MS/MS Analysis

Succinate, malonate and lactate were extracted from cells and tissues and analysed by LC-MS/MS as described in detail previously.[21,25,50] LC-MS/MS analysis of malonate and succinate was performed using an LCMS-8060 mass spectrometer (Shimadzu, UK) with a Nexera UHPLC system (Shimadzu, UK). Samples were stored in a refrigerated autosampler (4 °C) upon injection of 5 µl into a 15 µl flowthrough needle. Separation was achieved using a SeQuant ZIC-HILIC column (3.5 µm, 100 Å, 150 x 2.1 mm, 30 °C column temperature; Merck Millipore, UK) with

a ZIC-HILIC guard column (200 Å, 1 x 5mm). A flow rate of 200 µl/min was used with mobile phases of A) 10 mM ammonium bicarbonate (pH unchanged) and B) 100 % acetonitrile. A gradient of 0-0.1 min, 80% MS buffer B; 0.1-4 min, 80%-20% B; 4-10 min, 20% B, 10-11 min, 20%-80% B; 11-15 min, 80% B was used. The mass spectrometer was operated in negative ion mode with multiple reaction monitoring (MRM) and spectra were acquired using Labsolutions software (Shimadzu, UK), with compound quantities calculated from relevant standard curves in MS extraction buffer (50% (v/v) methanol, 30% (v/v) acetonitrile, 20% (v/v) MS-grade water) compared to 1 nmol of relevant internal standard (either [$^{13}C_3$]-malonate or [$^{13}C_4$]-succinate for malonate and succinate, respectively). Lactate results are expressed as relative abundance (peak area of lactate versus peak area of [$^{13}C_4$]-succinate). All mass spectrometry analyses were carried out blinded to the treatment conditions.

Cellular Uptake of Malonate

C2C12, H9c2 or HeLa cells were plated in 6-well plates (300,000 or 500,000 cells/well, respectively) and adhered overnight. The following day, the medium was replaced with Krebs buffer (116 mM NaCl, 4.7 mM KCl, 1.2 mM $MgSO_4.7H_2O$, 25 mM HEPES, 1.4 mM $CaCl_2$, 11 mM glucose; pH 7.4, 37 °C) and cells equilibrated for 30 min at 37 °C before changing the medium with the appropriate experimental condition at pH 7.4 (buffer as above) pH 6 or pH 8 Krebs buffer (116 mM NaCl, 4.7 mM KCl, 1.2 mM $MgSO_4.7H_2O$, 1.4 mM $CaCl_2$, 11 mM glucose, pH 6 - 25 mM MES, pH 8 - 25 mM Tricine, 37 °C). Parallel plates were incubated under the same conditions and used to measure protein levels by BCA assay (Thermo Fisher Scientific, UK). After incubation, cells and supernatant were extracted for LC-MS/MS. Cells were washed 4 times with ice-cold PBS before 500 µl MS extraction buffer containing MS internal standard was added to each well and incubated for 15 min on dry ice. Cells were scraped into microcentrifuge tubes and together with the supernatant samples were agitated (1,200 rpm, 15 min, 4 °C) before incubating at -20 °C for 1 hour. Samples were centrifuged (17,000 x g, 10 min, 4 °C), the supernatant transferred to a fresh tube and recentrifuged under the same conditions. The resulting supernatant was transferred to pre-cooled MS vials and analysed by LC-MS/MS.

MCT1 Knockdown

HeLa cells were plated at 100,000 cells/well in a 6-well plate and adhered overnight. 10 pmol MCT1 siRNA (1299001, Thermo Fisher, UK; sc-37235, Santa Cruz, UK) or control siRNA (12935300, Thermo Fisher, UK; sc-37007, Santa Cruz, UK) or MCT4 siRNA (h2, sc-45892, Santa Cruz, UK) were used to transfect with each well in 250 µl Optimem (Gibco, UK) containing 5 µl Lipofectamine RNAiMAX transfection reagent (Invitrogen, UK).

For C2C12 cells, 30,000 cells/well were plated and adhered overnight. 10 pmol MCT1 siRNA (MSS209081, #1320001, Thermo Fisher, UK; SASI_Mm01_00112354, #NM_009196, Merck, UK) or control siRNA (12935300, Thermo Fisher, UK) were used to transfect each well in 250 µl Optimem (Gibco, UK) containing 10 µl Lipofectamine RNAiMAX transfection reagent (Invitrogen, UK).

After 24 hours, the medium was replaced and the cells further cultured for 48 hours. Cells were subsequently used for analysis of MCT1 expression by Western blot or malonate uptake experiments.

Western Blot

Cells were directly lysed in 1 x Laemmli buffer (supplemented with 100 mM DTT and 1 µl/ml Benzonase endonuclease; 100 µl Laemmli buffer/well) and incubated on ice for 15 min before boiling. Tissues were lysed in 1 x Laemmli buffer (supplemented with 100 mM DTT and 1 µl/ml Benzonase endonuclease; 25 µl Laemmli buffer/mg tissue), centrifuged (17,000 x g, 10 min, 4 °C) and the supernatant boiled. Lysates (~20 µg protein) were loaded on 4-20% Tris-Glycine SDS-PAGE gels (Bio-Rad) and run at 180 V for ~45 min in SDS-PAGE running buffer (192 mM glycine, 25 mM Tris, 1% (w/v) SDS). The protein was transferred to PVDF membranes (Immobilon®-FL, Merck) by wet transfer (25 mM Tris, 192 mM glycine, 20% (v/v) methanol, pH (8.4)) before blocking for 1 h at RT with Odyssey blocking buffer (TBS; LICOR Biosciences). Membranes were incubated with primary antibodies in 4 % (v/v) Odyssey buffer in TBS + 0.1 % (v/v) Tween-20 (TBST) at 4 °C for 16 h or RT for 1 h. Membranes were then washed 3 x 5 min in TBST, followed by secondary incubation in 4 % (v/v) Odyssey buffer in PBS for 1 h at RT, washed 2 x 5 min in TBST and 1 x 5 min in TBS. Membranes were then visualised using a LICOR Odyssey CLx system and analysed using ImageStudio Lite. Primary antibodies: mouse monoclonal anti-MCT1 (H-1, sc-365501, Santa Cruz, 1:1,000), rabbit polyclonal anti-MCT1 (20139-1-

AP, Proteintech, 1:10,000), mouse monoclonal anti-MCT4 (G-7, sc-376465, Santa Cruz, 1:1,000), rabbit anti-vinculin (E1E9V, #13901, CST, 1:5,000), mouse anti-vinculin (Clone VIN-11-5, SAB4200729, Merck, 1:5,000). Secondary antibodies: goat anti-rabbit IgG (H+L) (DyLight™ 800 4X PEG Conjugate) (#5151, CST, 1:20,000) and goat anti-mouse IgG (H+L) (DyLight™ 680 Conjugate) (#5470, CST, 1:20,000).

Langendorff Isolated Heart Perfusions

Langendorff hearts were carried out as described in detail previously.[21] Mice were randomly assigned to treatment groups. Mice were administered terminal anaesthesia via intra-peritoneal pentobarbitone injection (~140 mg/kg body weight). Beating hearts were rapidly excised, cannulated and perfused in isovolumic Langendorff mode at 80 mm Hg pressure maintained by a STH peristaltic pump controller feedback system (AD Instruments, UK), with phosphate-free Krebs-Henseleit (KH) buffer continuously gassed with 95% O_2/5% CO_2 (pH 7.4, 37 °C) containing (in mM): NaCl (116), KCl (4.7), $MgSO_4.7H_2O$ (1.2), $NaHCO_3$ (25), $CaCl_2$ (1.4), glucose (11) or using the pH KH buffers described above.[51] Hearts were perfused for 20 min to equilibrate before being subjected to intervention protocols.

For IR malonate uptake studies, hearts were perfused for 20 min before inducing global no-flow ischemia for varying lengths of time before reperfusing for 5 min with 5 mM malonate (±10 or 50 µM AR-C141990 in the perfusion buffer where indicated). For pH studies, hearts were equilibrated for 20 min before infusing 5 mM malonate in pH 6 or 7.4 perfusion buffer for 5 min (± 50 mM lactate or 10 µM AR-C1441990 in the perfusion buffer where indicated). The hearts were flushed with non-malonate buffer before clamp freezing.

In vivo Left Anterior Descending (LAD) Coronary Artery Occlusion Model

The left anterior descending (LAD) coronary artery was occluded to induce MI in an acute open chest, in situ mouse model as described previously[52] to assess the effects of malonate on IR injury. Mice were randomly assigned to treatment groups and operators blinded to the treatments. Briefly, mice were anaesthetized by administration of sodium pentobarbital (70 mg/kg intraperitoneally), endotracheally intubated, ventilated with 3 cm H_2O positive end expiratory pressure and kept at 37 °C using a rectal thermometer-controlled heatpad (TCAT-2LV, Physitemp, USA). Ventilation frequency was maintained at 110 breaths/min, with tidal volume between

125 and 150 µl. The heart was exposed and a suture was placed around the prominent branch of the LAD and passed through a small plastic tube used to initiate ischemia by pressing the tube against the heart surface to occlude the LAD.

For infarct size determination: Mice were subjected to 30 min of ischemia and 120 min of reperfusion, after reperfusion, hearts were stained with Evans Blue and 2% triphenyltetrazolium chloride (TTC) and blindly analyzed by an independent researcher. Representative images were chosen based on a section representing the middle of the left ventricle in each of the conditions. DSM (160 mg/kg total dose in 0.9% saline) was infused over 20 min either during ischemia (starting 5 min before ischemia and continuing to infuse for 15 min during ischemia) or during reperfusion (starting 5 min before reperfusion and continuing the infusion for 15 min during reperfusion).

For experiments with CsA, all conditions were carried out using the CsA vehicle formulation[53] consisting of 27.8 mg/ml 94% ethanol and 65 mg/ml Cremophor EL (Kolliphor® EL, Sigma-Aldrich) in 0.9% saline and infused for 20 min during reperfusion.

For mass spectrometry imaging, hearts were subjected to the following groups: 30 min LAD occlusion, 30 min LAD occlusion and 15 min control reperfusion, 30 min LAD occlusion and 15 min reperfusion with 160 mg/kg DSM. After each experiment, the heart was rapidly excised and snap-frozen in liquid nitrogen-cool isopentane and stored at -80 °C until subsequent analysis.

Mass Spectrometry Imaging

All heart samples were co-embedded into a hydrogel-based multi-tissue block[54] and sections were collected at 10 µm thick sections using a Leica cryostat (Leica Biosystems, Germany). The sections were either thaw-mounted onto SuperFrost slides (Thermo Scientific, Germany) for MSI analysis or TOMO Adhesion Microscope Slides (Matsunami Glass Ind. Ltd., Japan) for histological stains. Sections were collected at different tiers from the apex upwards with an interval spacing of 0.5 mm. A total of 6 tiers were collected and analyzed to fully represent the metabolic heterogeneity within the tissues. The sections were air-dried and vacuum-packed for storage at –80 °C prior to MSI. MSI experiments were performed using a Q-Exactive plus mass spectrometer (Thermo Scientific, UK) with an automated desorption

electrospray ionization (DESI) ion source (Prosolia Inc., USA). Data were acquired in negative ion mode between m/z 80 and 1000. The nominal mass resolution was set to 70,000. The injection time was 150 ms, resulting in a scan rate of 3.8 pixel/s. A home-built DESI sprayer was operated with a mixture of 95% methanol/5% water at 1.5 µl/min and nebulized with nitrogen at a back pressure of 6 bar. The spatial resolution was 100 µm. Data analysis was performed in the SCiLS lab software package (V. 2020b, Bruker Daltonics, Germany) where the muscle tissue was delineated using a partial least squares machine learning classifier (PLS) based on manual annotations. The ischemic lesions were subsequently determined based on metabolic changes identified by principal component analysis (PCA) followed by bisecting k-Means clustering based on the most discriminant loadings of the relevant principal components. Sections collected for histological evaluation stained with hematoxylin and eosin (H&E) or silver infarct stain[55] and imaged on an Aperio scanner (Leica Biosystems, Germany) at 20x resolution. MSI data presented as the relative abundance of succinate which is the succinate peak area normalised to the total ion current. Representative images were chosen based on a section tier representing the middle of the left ventricle in each of the conditions.

Cellular Oxygen Consumption Measurements

Oxygen consumption of cells was measured using a Seahorse 96eXF Extracellular Flux Analyser (Agilent, UK). C2C12 mouse myoblasts or HeLa cells were plated in Seahorse 96-well plates (10,000 cells/well; Agilent) and adhered overnight in a humidified incubator at 37 °C and 5% CO_2. The following day, the media was replaced with Krebs buffer at pH 7.4 or 6 ± 5 mM DSM ± 10 µM AR-C141990 and incubated for 30 min before measuring oxygen consumption rate by a Seahorse XFe96 analyzer (Agilent, UK). Sequential port additions of oligomycin, FCCP, rotenone and antimycin A were used at final concentrations of 1.5 µM, 1 µM, 4 µg/ml and 10 µM respectively.

H_2O_2 measurement with MitoB

The measurement of ROS production in reperfused at-risk tissue was assessed using MitoB and its conversion to MitoP as described previously.[18,56] Briefly, mice were randomised and were prepared for the in-situ LAD ligation model and 75 nmol MitoB injected IV as a 100 µl bolus 5 min before the induction of ischemia. Mice were

subjected to 30 min ischemia and reperfused for 15 min with saline or DSM (160 mg/kg) infused for 20 min starting 5 min before reperfusion (operator blinded to treatment conditions). The at-risk tissue was excised and clamp frozen in liquid nitrogen before processing and measuring MitoB and MitoP by LC-MS/MS with the operator blinded to treatment groups, with quantification by interpolating a standard curve.[56]

Statistical Analysis

Statistical analysis was carried out using Prism 9.1 software (Graphpad Software Inc, USA). Normality was assessed using the Shapiro-Wilk test. Statistical analysis was performed using one-way ANOVA, Kruskal-Wallis test or Friedman's test with the suitable correction for multiple comparisons or one or two-tailed unpaired Mann-Whitney U test or Student's t-test (summary of statistical tests provided at end of supplementary material). A p-value of 5.00×10^{-2} was considered significant. In all instances, biological replicates were used for statistical analyses; where technical replicates were performed (all in vitro studies used at least 2 technical replicates per experiment), these were averaged to represent one biological replicate and the whole experiment performed multiple times. Where appropriate, analyses were carried out in a randomized and blinded fashion.

Summary of statistical tests

Figure 1

C) Kruskal-Wallis with Dunn's post hoc test of infarct size with malonate treatment vs control

D) Kruskal-Wallis with Dunn's post hoc test of infarct size with malonate treatment vs control

E) Kruskal-Wallis with Dunn's post hoc test of malonate levels in non-risk vs at-risk tissue

F) Two-way ANOVA with Tukey's post hoc test of succinate levels in non-risk and at risk-tissue with or without malonate treatment, all comparisons made

G) Unpaired, two-tailed Mann-Whitney U test of MitoP/B ratio in at-risk tissue of control vs malonate treatment

H) Kruskal-Wallis with Dunn's post hoc test of infarct size with treatments vs control and CsA vs CsA + malonate

Figure 2

C) Friedman's paired test with Dunn's post hoc test for succinate abundance healthy vs lesion and Kruskal-Wallis with Dunn's post hoc test for succinate abundance in lesion area, all comparisons made

Figure 3

A) Kruskal-Wallis with Dunn's post hoc test of malonate levels at different pH vs pH 6, separate test for each malonate concentration

B) Kruskal-Wallis with Dunn's post hoc test of succinate levels at different pH vs pH 6, separate test for each malonate concentration

D) Unpaired, two-tailed Mann-Whitney U test

E) Kruskal-Wallis with Dunn's post hoc test of malonate levels at different FCCP concentrations vs control (0), separate test for each pH

F) Kruskal-Wallis with Dunn's post hoc test of malonate levels at different Gramicidin concentrations vs control (0), separate test for each pH

G) Kruskal-Wallis with Dunn's post hoc test of malonate levels at different Nigericin concentrations vs control (0), separate test for each pH

H) Kruskal-Wallis with Dunn's post hoc test of malonate levels at different BAM15 concentrations vs control (0), separate test for each pH

J) Unpaired, two-tailed Student's t-test test

K) Unpaired, two-tailed Student's t-test test

Figure 4

A) Kruskal-Wallis with Dunn's post hoc test of malonate levels at different lactate concentrations vs control (0), separate test for each pH

G) Kruskal-Wallis with Dunn's post hoc test of OCR with treatments vs control (vehicle), separate test for each respiration state

H) Kruskal-Wallis with Dunn's post hoc test of OCR with treatments vs control (vehicle), separate test for each respiration state

Figure 5

A) one way-ANOVA with Bonferroni's post hoc test of treatment vs control (Ctl)

B) Kruskal-Wallis with Dunn's post hoc test of malonate levels with different siRNA vs control (Ctl siRNA), separate test for each condition

C) Kruskal-Wallis with Dunn's post hoc test of malonate levels with different siRNA vs control (Ctl siRNA), separate test for each condition

D) Kruskal-Wallis with Dunn's post hoc test of malonate levels with different siRNA vs control (Ctl siRNA), separate test for each condition

H) Unpaired, two-tailed Mann-Whitney U test versus control

Figure 6

A) Kruskal-Wallis with Dunn's post hoc test vs 0 min ischemia

B) Unpaired, two-tailed Mann-Whitney U test versus control

D) Unpaired, two-tailed Mann-Whitney U test

E) Unpaired, two-tailed Mann-Whitney U test versus acid malonate

Supplementary Figure 1

A) Kruskal-Wallis with Dunn's post hoc test of malonate levels at different malonate concentrations vs control

B) Kruskal-Wallis with Dunn's post hoc test of succinate levels at different malonate concentrations vs control

D) Kruskal-Wallis with Dunn's post hoc test of malonate levels at different malonate concentrations vs control

E) Kruskal-Wallis with Dunn's post hoc test of succinate levels at different malonate concentrations vs control

Supplementary Figure 2

A) Kruskal-Wallis with Dunn's post hoc test of malonate levels at different pH vs pH 6, separate test for each malonate concentration

B) Kruskal-Wallis with Dunn's post hoc test of succinate levels at different pH vs pH 6, separate test for each malonate concentration

Supplementary Figure 3

A) Kruskal-Wallis with Dunn's post hoc test of malonate levels at different Monensin concentrations vs control (0), separate test for each pH

B) Kruskal-Wallis with Dunn's post hoc test of succinate levels at different FCCP concentrations vs control (0), separate test for each pH

C) Kruskal-Wallis with Dunn's post hoc test of succinate levels at different Gramicidin concentrations vs control (0), separate test for each pH

D) Kruskal-Wallis with Dunn's post hoc test of succinate levels at different Nigericin concentrations vs control (0), separate test for each pH

E) Kruskal-Wallis with Dunn's post hoc test of succinate levels at different Monensin concentrations vs control (0), separate test for each pH

F) Kruskal-Wallis with Dunn's post hoc test of succinate levels at different BAM15 concentrations vs control (0), separate test for each pH

G) Kruskal-Wallis with Dunn's post hoc test of malonate levels at different DIDS concentrations vs control (0), separate test for each pH

H) Kruskal-Wallis with Dunn's post hoc test of succinate levels at different DIDS concentrations vs control (0), separate test for each pH

Supplementary Figure 4

A) Kruskal-Wallis with Dunn's post hoc test of succinate levels at different lactate concentrations vs control (0), separate test for each pH

Supplementary Figure 5

B) Unpaired, two-tailed Mann-Whitney U test versus control

D) Unpaired, one-tailed Mann-Whitney U test of siRNA vs relevant control

E) Kruskal-Wallis with Dunn's post hoc test of malonate levels of MCT1 siRNA vs relevant siRNA control, separate test for each condition

G) Kruskal-Wallis with Dunn's post hoc test of MCT4 levels vs control

Supplementary Figure 6

A) Unpaired, one-tailed Mann-Whitney U test

C) One way-ANOVA with Bonferroni's post hoc test, all comparisons made

DATA SUPPLEMENT FIGURES

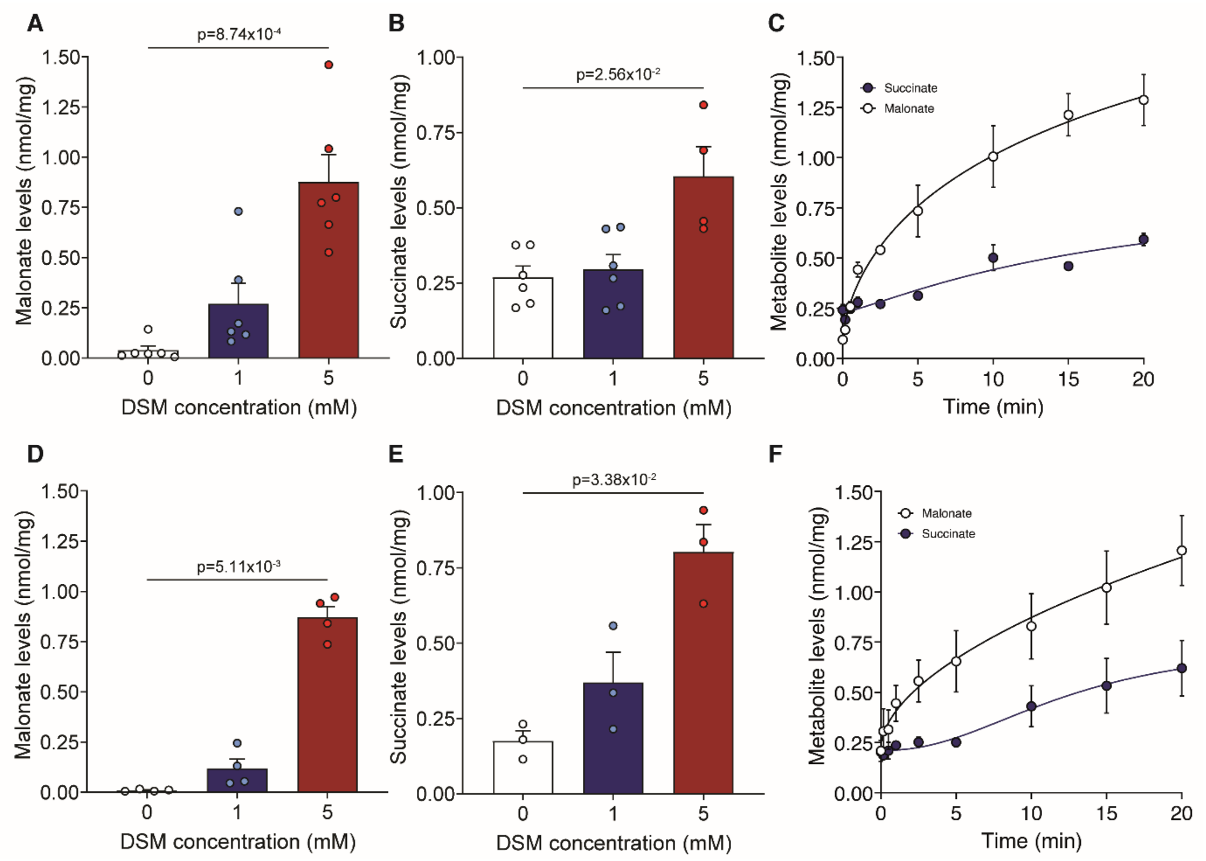

Supplementary Figure 1. Malonate uptake in C2C12 and H9c2 cells. C2C12 (**A** to **C**) and H9c2 cells (**D** to **F**) were incubated with DSM (0, 1 or 5 mM) for 15 min before measuring intracellular malonate (**A** and **D**) and succinate (**B** and **E**) by LC-MS/MS (statistics: Kruskal-Wallis with Dunn's post hoc test). **C** and **F**, C2C12 (**C**) or H9c2 (**F**) cells were treated with 5 mM DSM for 0-20 min and intracellular malonate and succinate measured (mean ± S.E.M, n=3 (**E, C, F**), 4 (**D** and 5 mM DSM in **B**) or 6 (**A** and 0 and 1 mM in **B**).

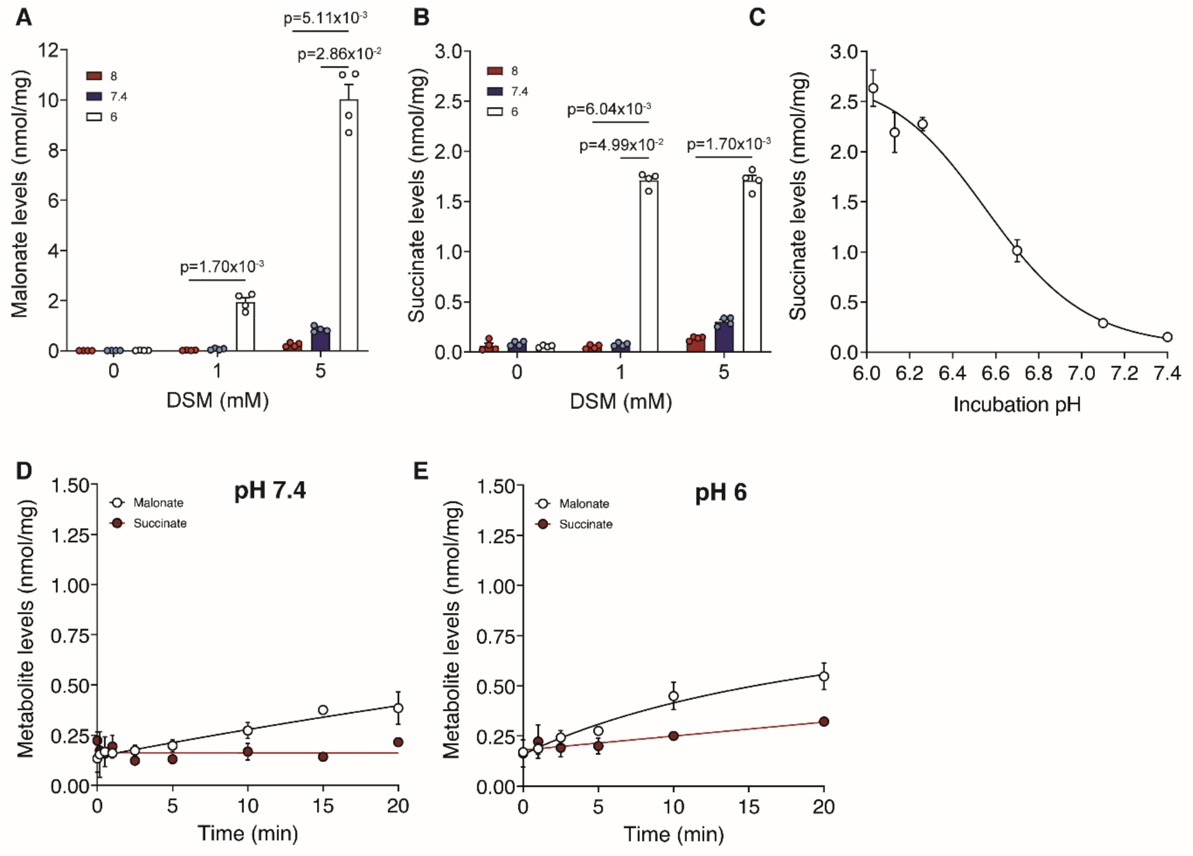

Supplementary Figure 2. Low pH enhances malonate uptake

A and **B**, H9c2 cells were incubated with DSM (0, 1 or 5 mM) for 15 min at either pH 6, 7.4 or 8 before measuring intracellular malonate (**A**) and succinate (**B**) by LC-MS/MS (mean ± S.E.M, n=4 biological replicates, statistics: Kruskal-Wallis with Dunn's post hoc test). **C**, Succinate levels in C2C12 cells incubated with DSM (5 mM) for 15 min at various pH (mean ± S.E.M, n=3 biological replicates). **D** and **E**, Malonate and succinate levels in C2C12 cells incubated with DSM (5 mM) from 0 to 20 min at pH 7.4 (**D**) or 6 (**E**) at 0 °C (mean ± S.E.M, n=3 biological replicates).

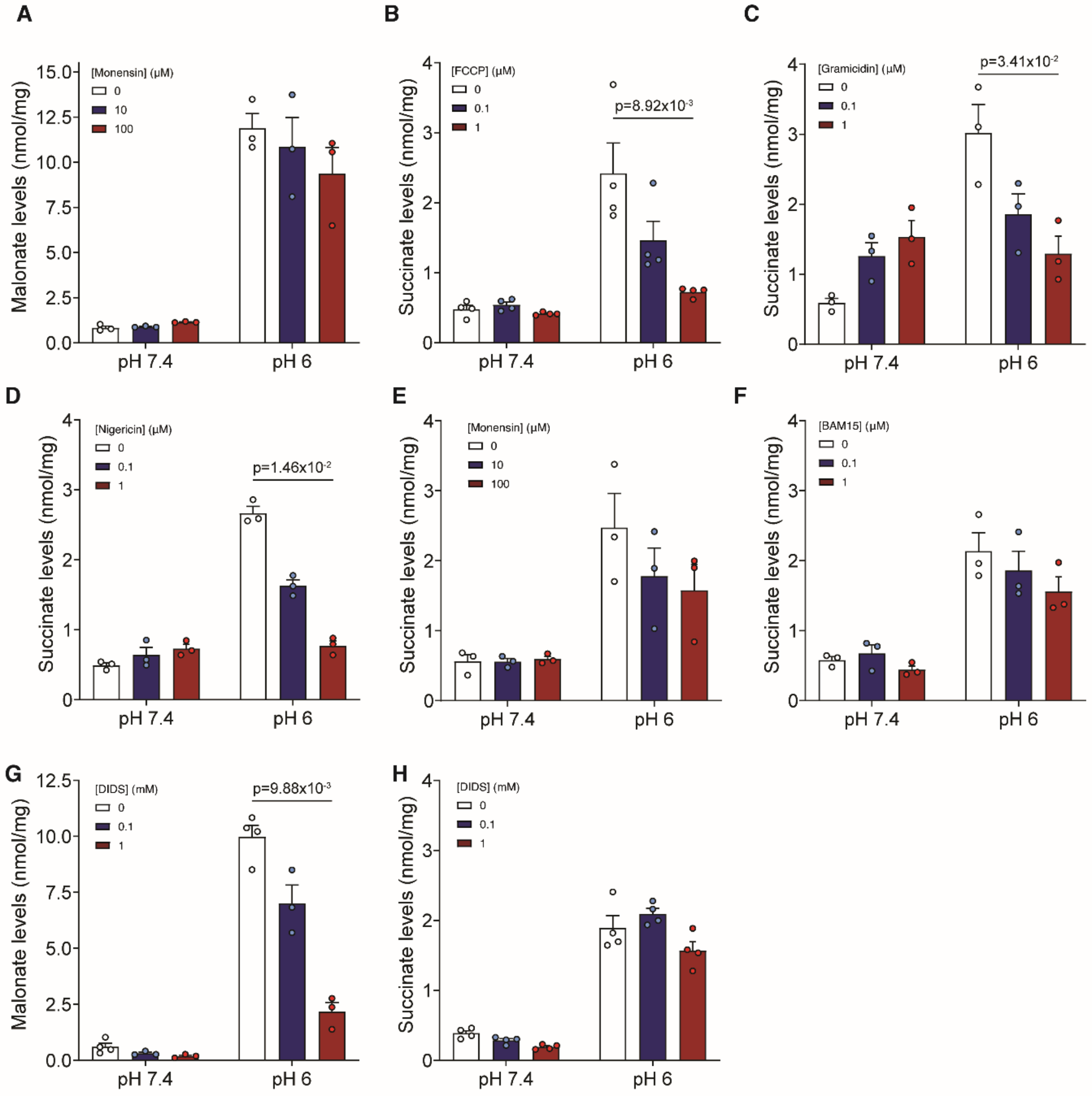

Supplementary Figure 3. Perturbing malonate uptake with ionophores.

A, Malonate levels in C2C12 cells incubated with DSM (5 mM) for 15 min at either pH 6 or 7.4 in the presence of monensin. **B** to **F**, Succinate levels in C2C12 cells incubated with DSM (5 mM) for 15 min at either pH 6 or 7.4 in the presence of FCCP (**B**), gramicidin (**C**), nigericin (**D**), monensin (**E**), BAM15 (**F**). **G** and **H**, Malonate (**G**) and succinate (**H**) levels in C2C12 cells incubated with DSM (5 mM) for 15 min at either pH 6 or 7.4 in the presence of DIDS (mean ± S.E.M, n=3 (**A, C, D, E, F**) or 4 (**B, G, H**) biological replicates, statistics: Kruskal-Wallis with Dunn's post hoc test).

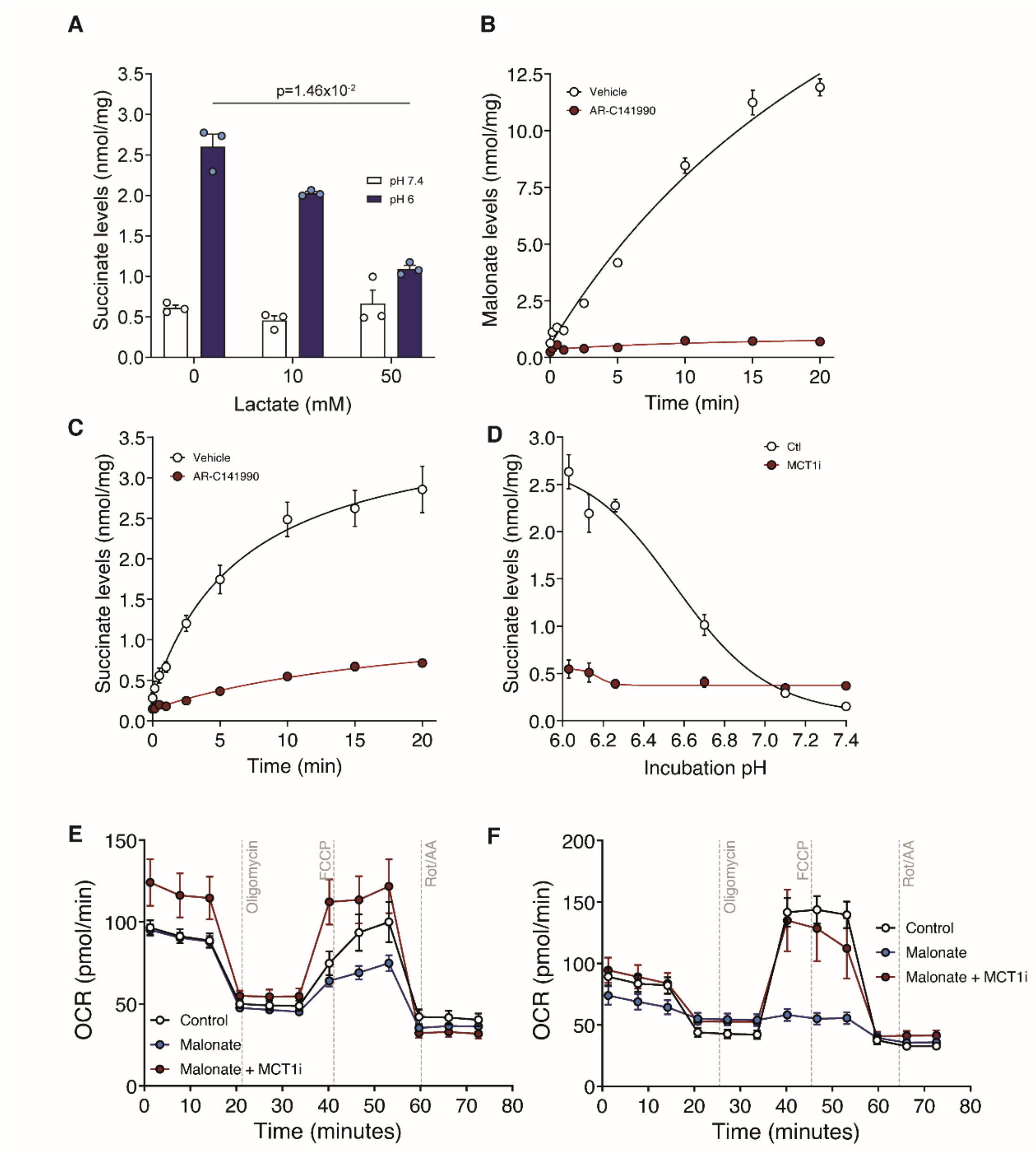

Supplementary Figure 4. Inhibition of MCT1 prevents succinate accumulation by reduced malonate uptake at lowered pH. A, Succinate levels in C2C12 cells treated with DSM (5 mM, 15 min) in the presence of excess lactate (0, 10 or 50 mM). (mean ± S.E.M., n=3 or 4 biological replicates, statistics: Kruskal-Wallis with Dunn's post hoc test). **B** and **C**, Time course of malonate uptake (**B**) and succinate levels (**C**) after C2C12 cell treatment with malonate (5 mM DSM) at pH 6 ± 10 µM AR-C141990. **D**, Succinate levels in C2C12 cells treated with malonate (5 mM DSM, 15 min) at various pH ± 10 µM AR-C141990 (mean ± S.E.M., n=3). **E** and **F**, Representative traces of oxygen consumption measurements in C2C12 cells ± 5 mM DSM or 10 µM AR-C141990 (MCT1i) at pH 7.4 (**E**) or pH 6 (**F**) in the absence of

inhibitors (baseline) or presence of 1.5 µM oligomycin, 1 µM FCCP or 4 µg/ml rotenone and 10 µM antimycin A. (data is presented as mean ± S.E.M., 12-16 technical replicates).

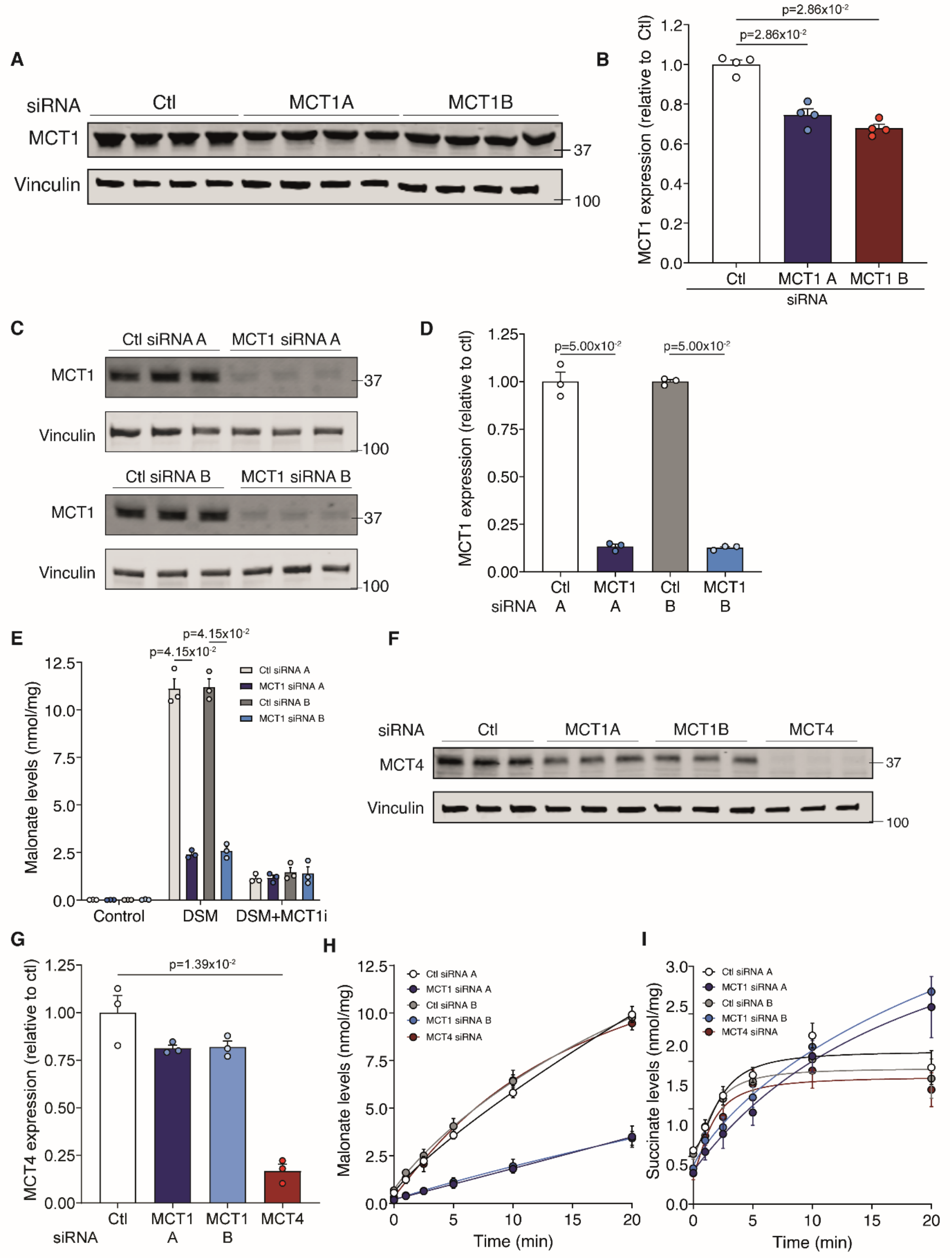

Supplementary Figure 5. Genetic knockdown of MCT1 but not MCT4 prevents the enhanced uptake of malonate at lowered pH in HeLa cells. A and **B**, Western

blot of MCT1 knockdown in C2C12 cells (**A**) and quantification (**B**) (Quantification is presented as mean ± S.E.M., 4 biological replicates, statistics: unpaired, two-tailed Mann-Whitney U test versus control). **C** and **D**, Western blot of MCT1 knockdown in HeLa cells (**C**) and quantification (**D**) (quantification is presented as mean ± S.E.M., 3 biological replicates, statistics: unpaired, one-tailed Mann-Whitney U test). **E**, Incubation of control (saline) or 5 mM DSM in MCT1 KD cells at 6 for 15 min ± MCT1i (10 µM AR-C141990) (mean ± S.E.M., n=3 biological replicates, statistics: Kruskal-Wallis with Dunn's post hoc test). **F** and **G**, Western blot of MCT4 knockdown in HeLa cells (**F**) and quantification (**G**) (quantification is presented as mean ± S.E.M., 3 biological replicates, statistics: Kruskal-Wallis with Dunn's post hoc test). **H** and **I**, Malonate uptake (5 mM DSM) at pH 6 time course in MCT1 or MCT4 KD cells (**H**) and corresponding succinate levels (**I**) (mean ± S.E.M., n=3 biological replicates).

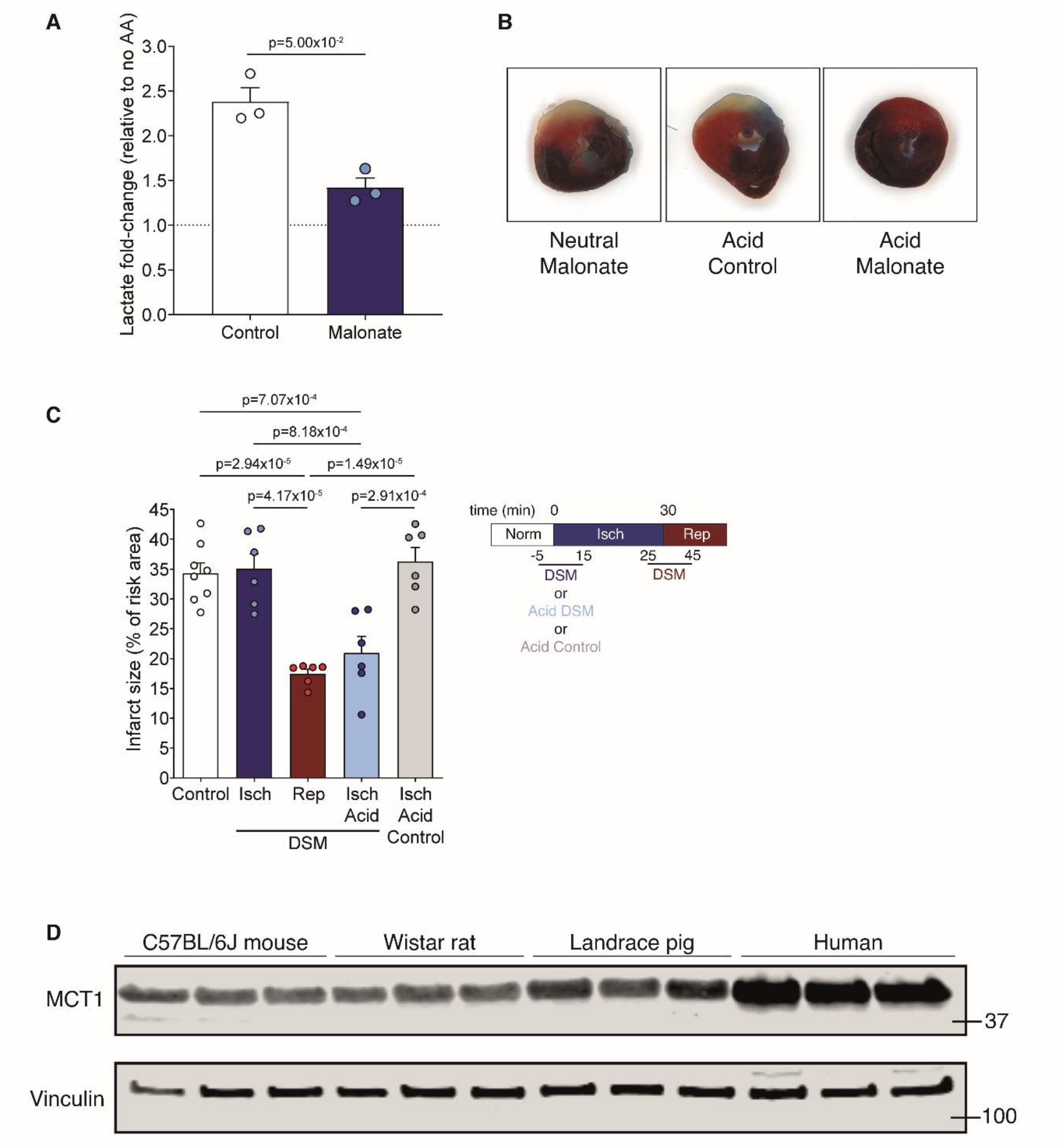

Supplementary Figure 6. MCT1 lactate/malonate exchange and expression in the human heart. **A,** Lactate abundance in C2C12 cells treated with or without 10 µM antimycin A for 15 min prior to treatment with 5 mM DSM or control (saline) for 15 min at pH 7.4 (mean ± S.E.M. of the fold change in lactate abundance with or without antimycin A, n=3 biological replicates, statistics: unpaired, one-tailed Mann-Whitney U test). **B**, Representative infarct images from murine LAD occlusion MI model with bolus of 8 mg/kg DSM, 8 mg/kg acid malonate or acid control. **C**, Infarct size in murine LAD model with infusion of neutral or acidic DSM (160 mg/kg) or acid control before ischemia or at reperfusion and quantified by TTC staining (data

compared with that from **Figure 1C**) (mean ± S.E.M., n=6 or 8 (control) biological replicates, statistics: one way-ANOVA with Bonferroni’s post hoc test). **D**, Western blot of mouse, rat, pig, human heart tissue MCT1 expression in heart tissue (n=3 biological replicates for each species – each lane a biological replicate).
