# Supplementary material for: Ischemia-Selective Cardioprotection by Malonate for Ischemia/Reperfusion Injury
Source: Circ Res. Author manuscript; Available in PMC 2022 Sep 9. (PMC9426742; doi:10.1161/CIRCRESAHA.121.320717)

Full unedited gel for Supplementary Figure 5A

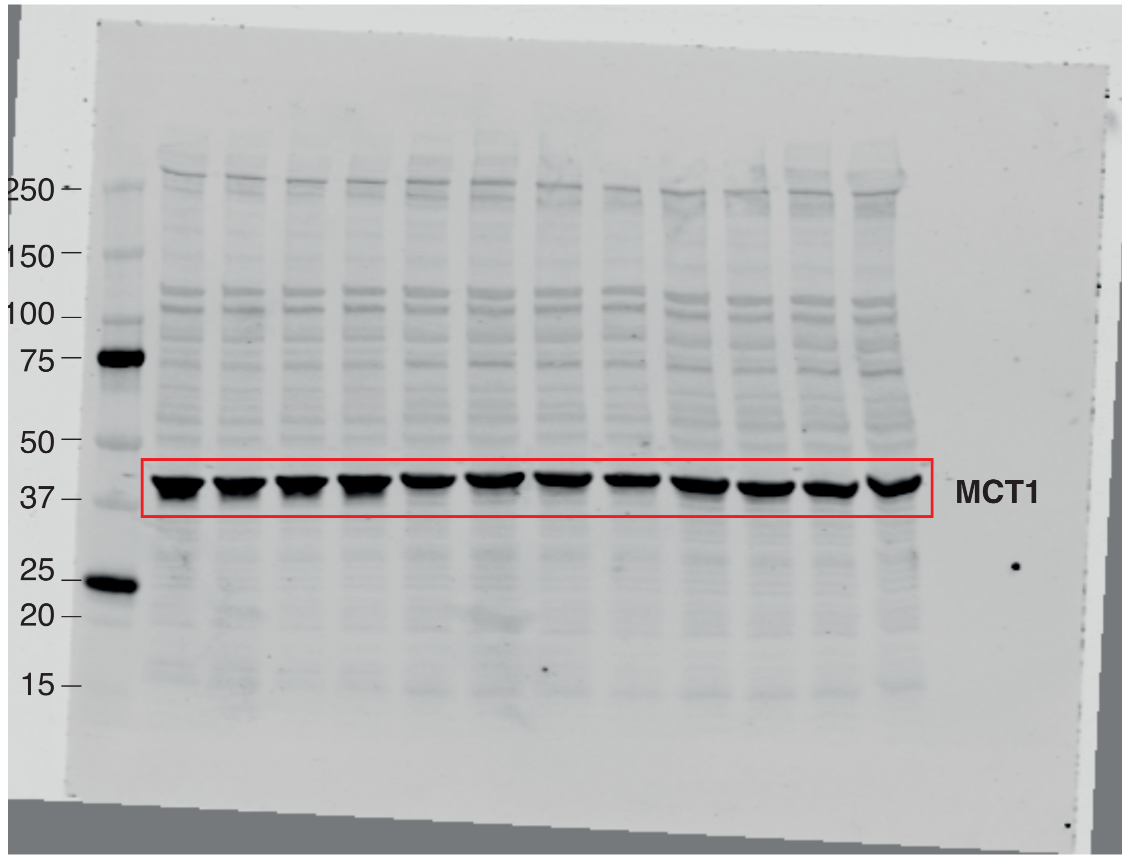

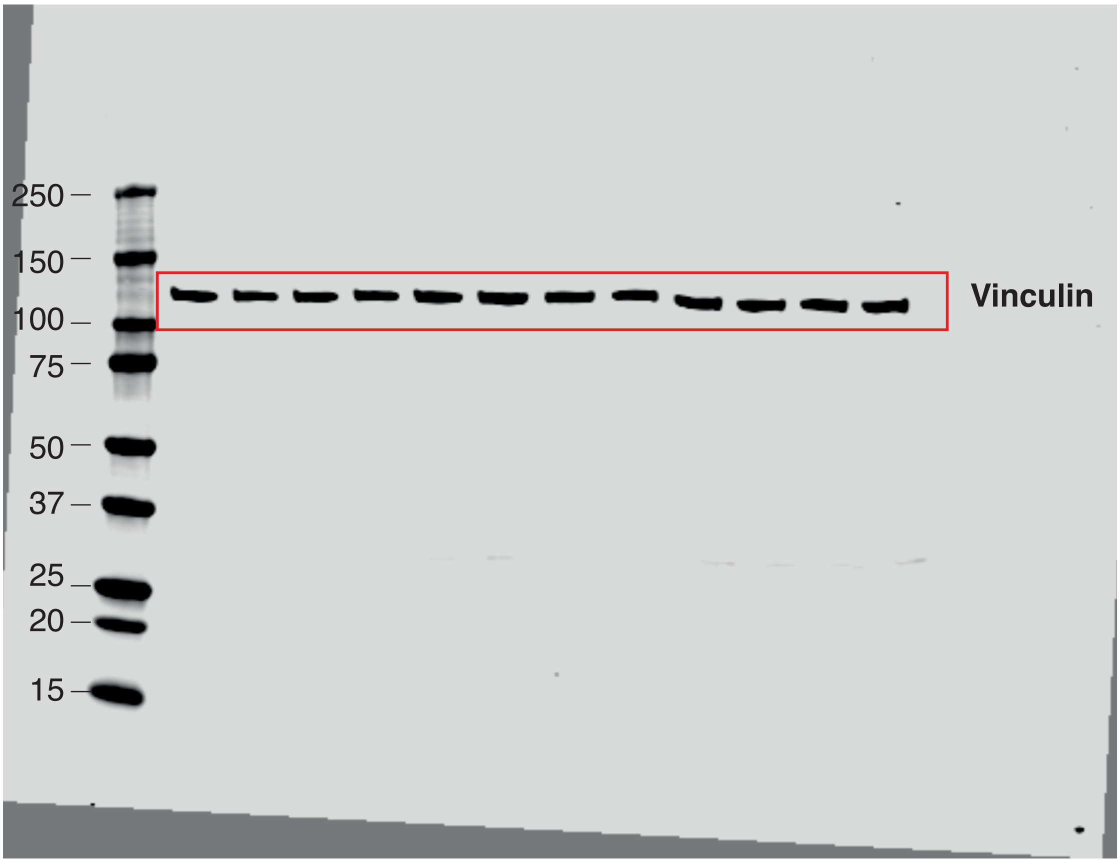

Full unedited gel for Supplementary Figure 5C

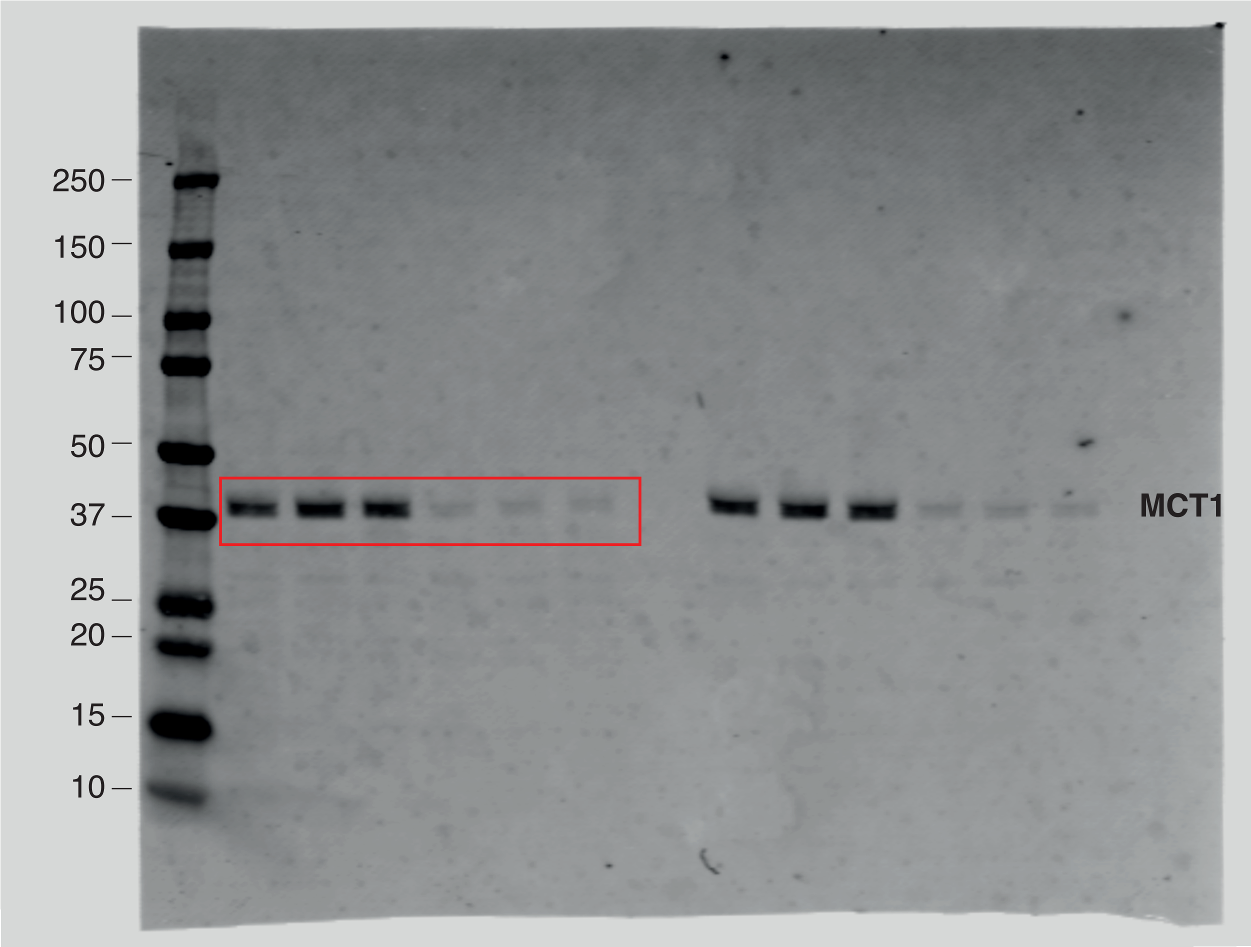

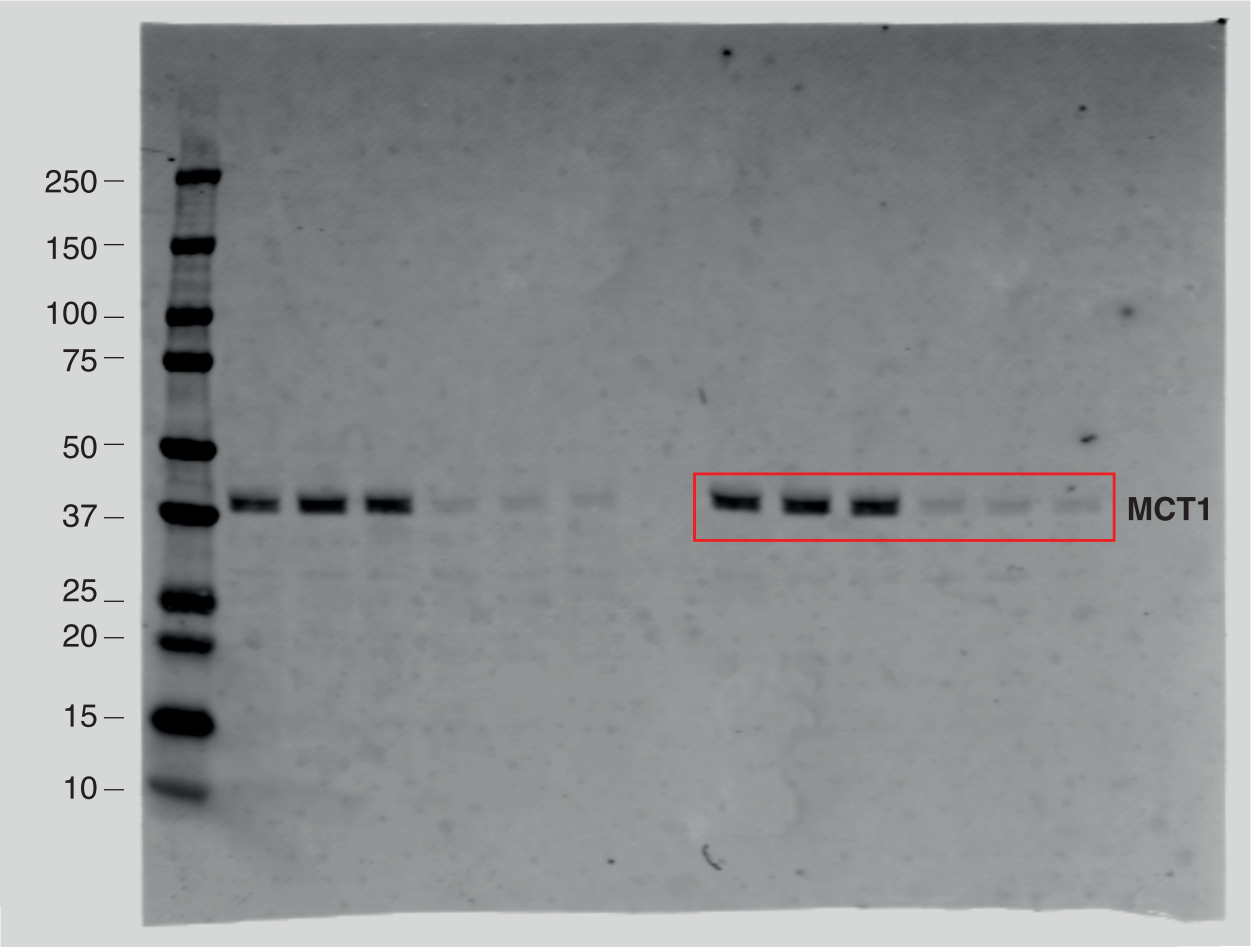

Full unedited gel for Supplementary Figure 5C

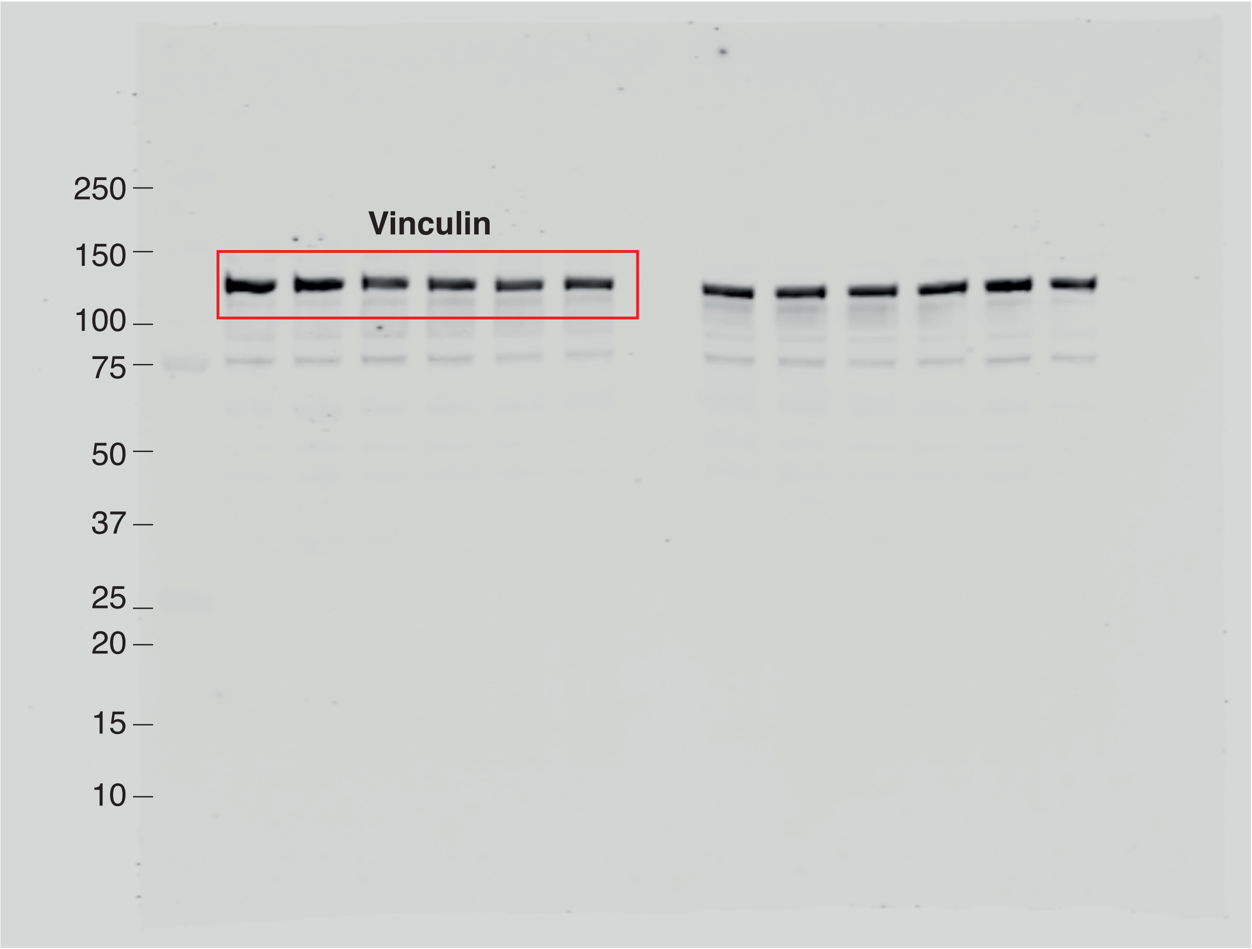

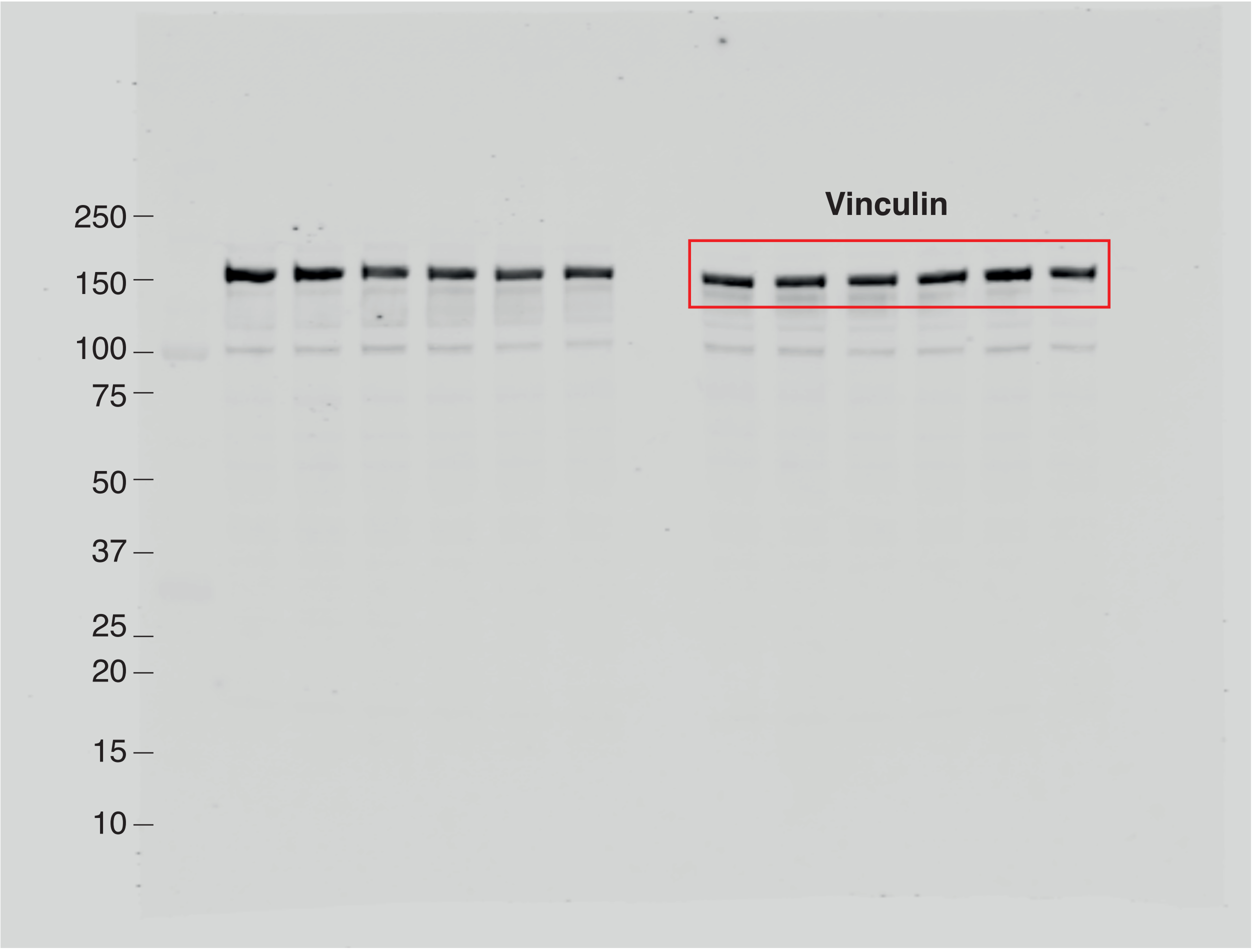

Full unedited gel for Supplementary Figure 5F

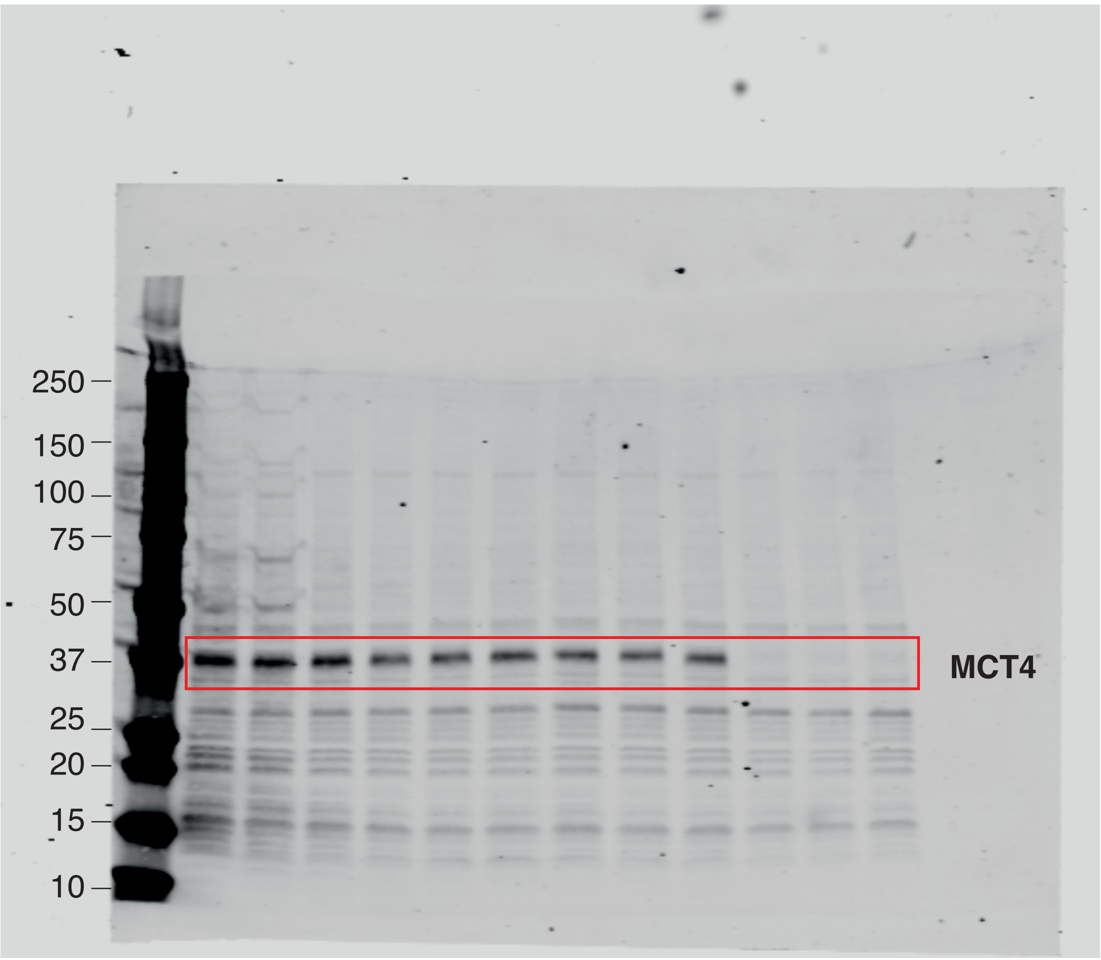

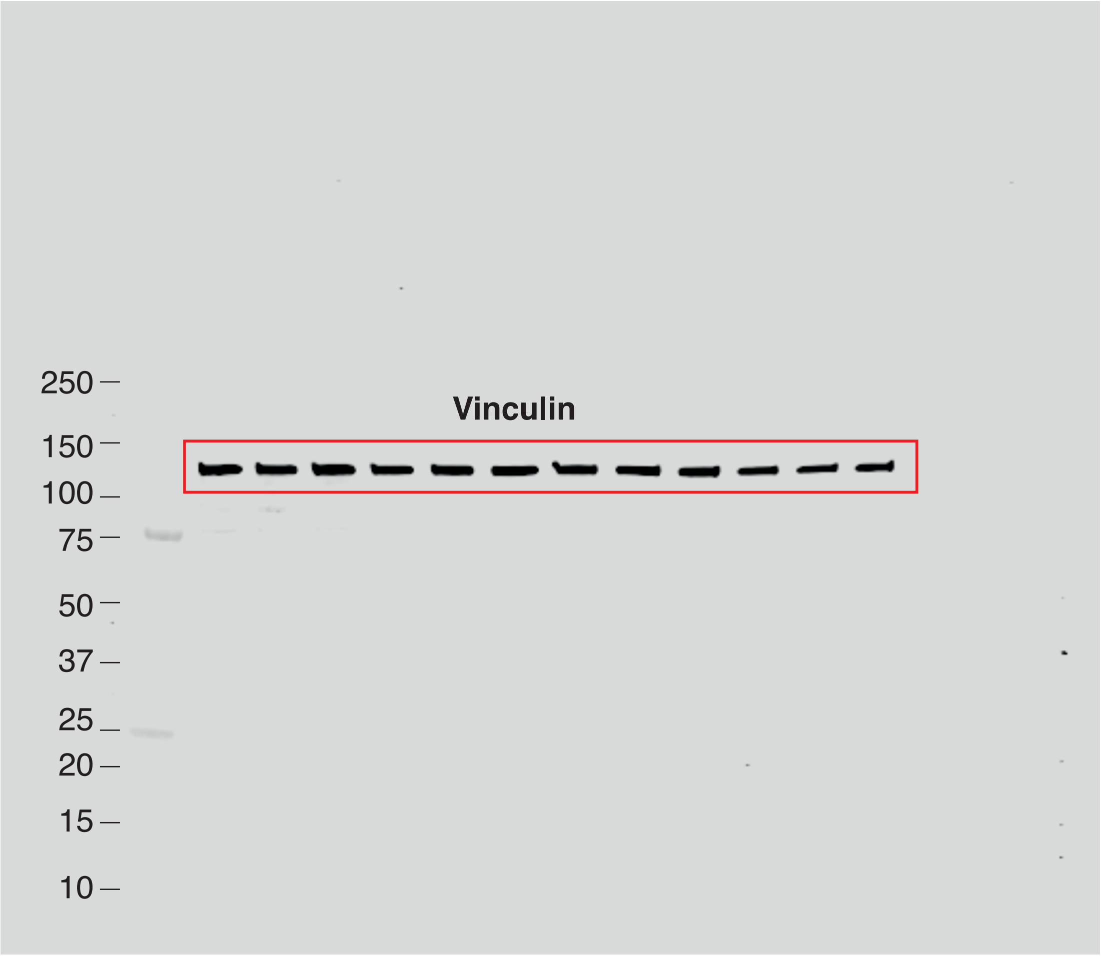

Full unedited gel for Supplementary Figure 6D

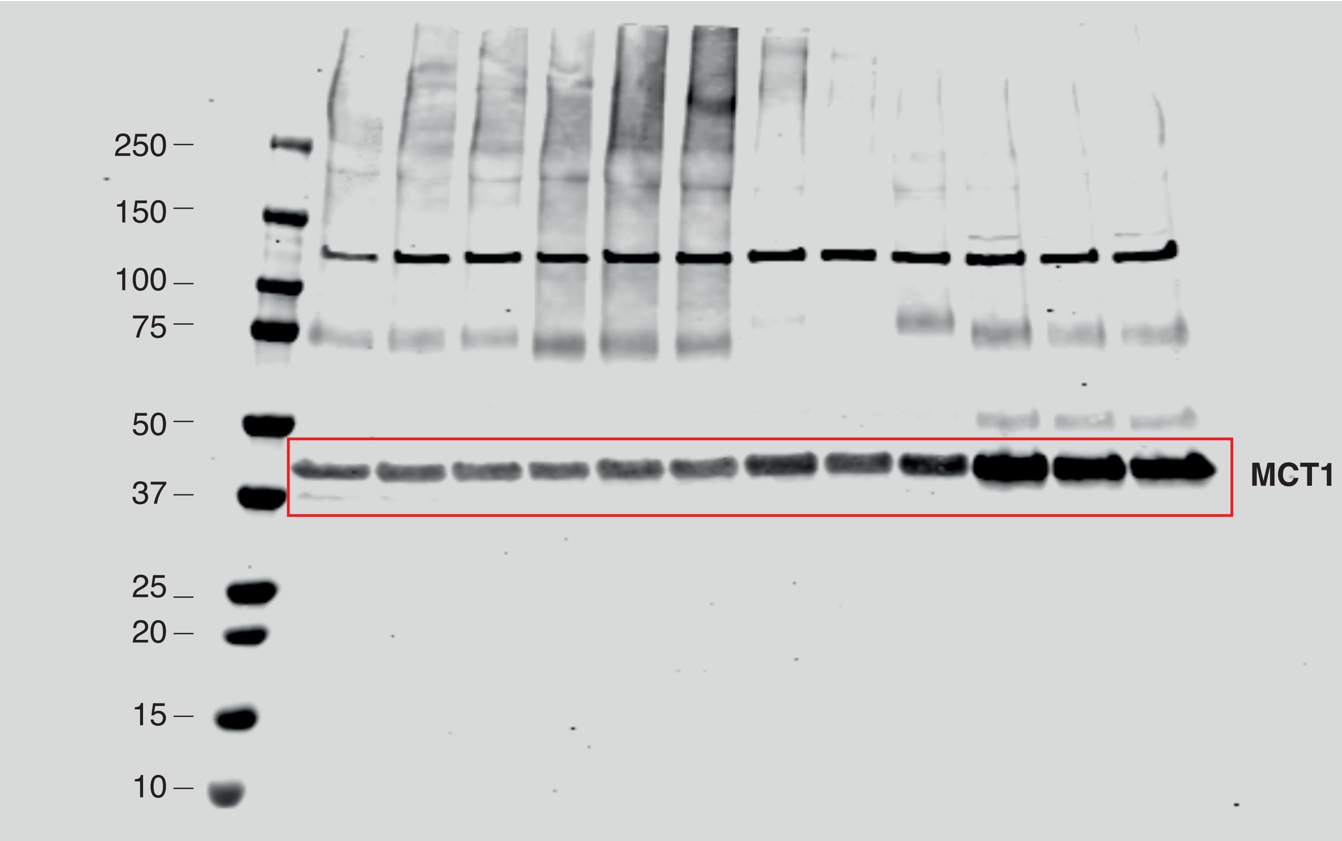

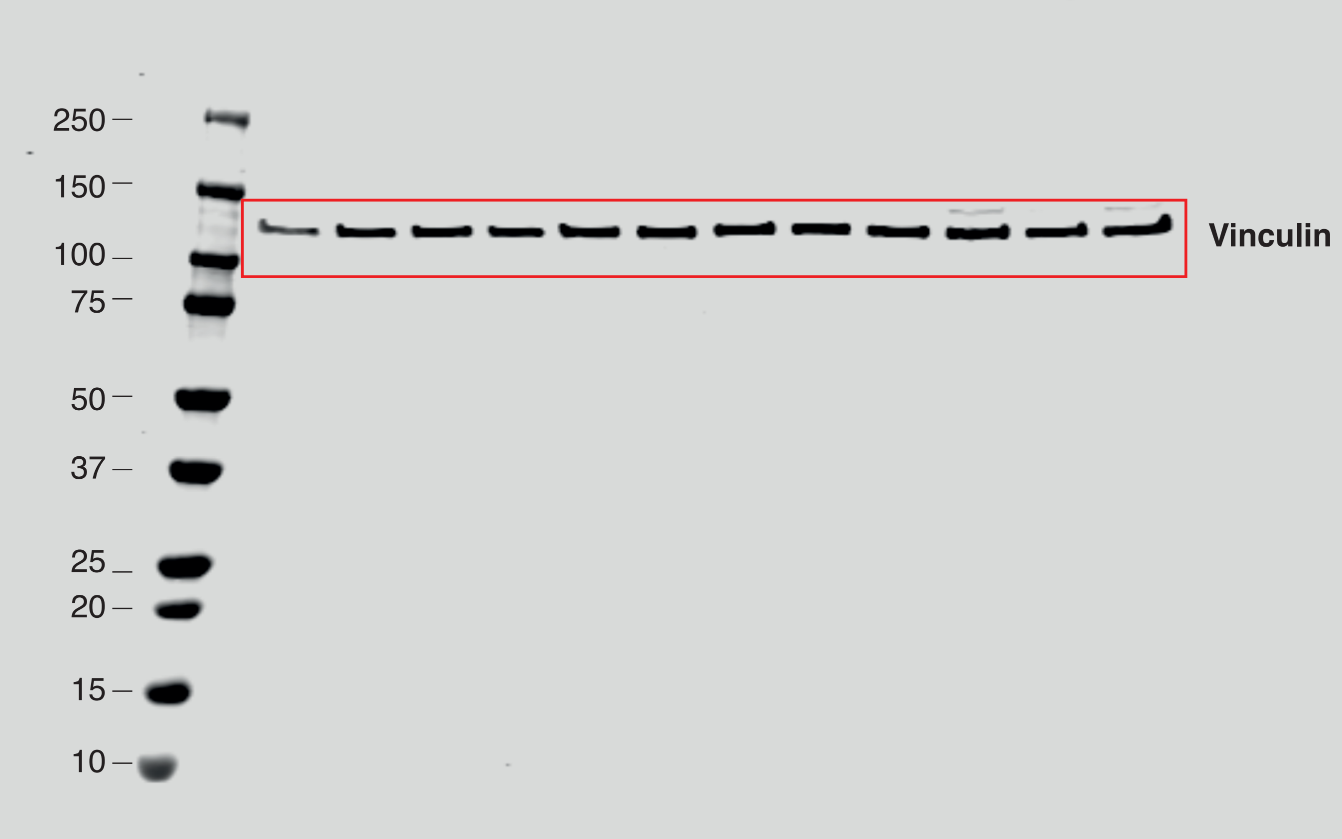

Supplement: Uncut Gel Blots [file EMS152108-supplement-Uncut_Gel_Blots.pdf]
