# Supplementary material for: Ischemia-Selective Cardioprotection by Malonate for Ischemia/Reperfusion Injury
Source: Circ Res. Author manuscript; Available in PMC 2022 Sep 9. (PMC9426742; doi:10.1161/CIRCRESAHA.121.320717)
Supplement: Major Resources Table [file EMS152108-supplement-Major_Resources_Table.pdf]

Major Resources Table

In order to allow validation and replication of experiments, all essential research materials listed in the Methods should be included in the Major Resources Table below. Authors are encouraged to use public repositories for protocols, data, code, and other materials and provide persistent identifiers and/or links to repositories when available. Authors may add or delete rows as needed.

Animals (in vivo studies)

Species	Vendor or Source	Background Strain	Sex	Persistent ID / URL
Mouse (*Mus musculus*)	Charles River or Laboratory Animal Science, Chinese Academy of Medical Sciences	C57BL/6J	Male	Cat#632 (Charles River)

Antibodies

Target antigen	Vendor or Source	Catalog #	Working concentration	Lot # (preferred but not required)	Persistent ID / URL
MCT1	Santa Cruz	H-1, sc-365501	1:1000		https://www.scbt.com/p/mct1-antibody-h-1
MCT1	Proteintech	20139-1-AP	1:10,000		https://www.ptglab.com/products/MCT1-Antibody-20139-1-AP.htm
MCT4	Santa Cruz	G-7, sc-376465	1:1,000		https://www.scbt.com/p/mct4-antibody-g-7
Vinculin	Merck	Clone VIN-11-5, SAB4200729	1:5,000		https://www.sigmaaldrich.com/GB/en/product/sigma/sab4200729
Vinculin	Cell Signaling Technology	E1E9V, 13901	1:5,000		https://www.cellsignal.co.uk/products/primary-antibodies/vinculin-e1e9v-xp-rabbit-mab/13901
Goat anti-rabbit IgG (H+L) (DyLight™ 800 4X PEG Conjugate)	Cell Signaling Technology	5151	1:20,000		https://www.cellsignal.co.uk/products/secondary-antibodies/anti-rabbit-igg-h-l-dylight-800-4x-peg-conjugate/5151
Goat anti-mouse IgG (H+L) (DyLight™ 680 Conjugate)	Cell Signaling Technology	5470	1:20,000		https://www.cellsignal.co.uk/products/secondary-antibodies/anti-mouse-igg-h-l-dylight-680-conjugate/5470

Cultured Cells

Name	Vendor or Source	Sex (F, M, or unknown)	Persistent ID / URL
C2C12 mouse myoblasts	ATCC	unknown	CRL-1772
H9c2 (2-1)	ATCC	unknown	CRL-1446
HeLa	ATCC	Female	CCL-2

DOI [to be added]

Other

Description	Source / Repository	Persistent ID / URL
Sodium malonate dibasic	Merck	63409
Cyclosporine A (CsA)	Merck	30024
Carbonyl Cyanide p-(Trifluoromethoxy)phenylhydrazone (FCCP)	Merck	C2920
Gramicidin from *Bacillus aneurinolyticus (Bacillus brevis)*	Merck	G5002
Nigericin sodium salt	Merck	N7143
BAM15	Merck	SML1760
Monensin sodium salt	Merck	M5273
Oligomycin from *Streptomycin diastatochromogenes*	Merck	O4876
Rotenone	Santa Cruz	sc-203242
Antimycin A from *Streptomyces sp.*	Merck	A8674
4,4′-Diisothiocyanatostilbene-2,2′-disulfonic acid (DIDS) disodium salt hydrate	Merck	D3514
Malonamic acid	Merk	S937436
Sodium L-Lactate	Merck	L7022
AZD3965	Cayman Chemical	19912
AR-C141990 hydrochloride	Biotechne (Tocris)	5658
Lipofectamine RNAiMAX	Invitrogen	13778150
Human MCT1 siRNA A	Thermo Fisher	1299001
Human MCT1 siRNA B	Santa Cruz	sc-37235
Control siRNA A	Thermo Fisher	12935300
Control siRNA B	Santa Cruz	sc-37007
Human MCT4 siRNA	Santa Cruz	H2, sc-45892
Mouse MCT1 A siRNA	Thermo Fisher	MSS209081, #1320001
Mouse MCT1 B siRNA	Merck	SASI_Mm01_00112354, #NM_009196
DMEM (high glucose, GlutaMAX, pyruvate)	Gibco	31966047
Fetal Bovine Serum	Gibco	10270106
Opti-MEM	Gibco	31985070
[$^{13}C_3$]-malonate	Merck	490202
[$^{13}C_4$]-succinate	Merck	491985
Kolliphor® EL	Merck	C5135
MitoB	Cayman Chemical	17116

ARRIVE GUIDELINES

The ARRIVE guidelines (https://arriveguidelines.org/) are a checklist of recommendations to improve the reporting of research involving animals. Key elements of the study design should be included below to better enable readers to scrutinize the research adequately, evaluate its methodological rigor, and reproduce the methods or findings.

Study Design

Groups	Sex	Age	Littermates (Yes/No)	Other description
All animals for in vivo LAD ligation experiments and ex vivo Langendorff	M	8-12 weeks	No	All were non-recovery experiments

Sample Size: Please explain how the sample size was decided Please provide details of any a *prior* sample size calculation, if done.

Sample sizes for in vivo experiments were based on previous power calculations and experience in the in vivo LAD ligation model of MI and ex vivo Langendorff perfusion model.

Inclusion Criteria

N/A

Exclusion Criteria

The exclusion criteria for LAD ligation was an area at risk out of the range of 30%-60%.

Randomization

Animals were randomly assigned to treatment or control groups.

Blinding

Investigators carrying out in vivo work were blinded to treatments and analysis was carried out blindly to reduce bias.

DOI [to be added]
